# Hydrophobic Aerogels and Xerogels based on Trimethoxybenzene‐Formaldehyde

**DOI:** 10.1002/marc.202400691

**Published:** 2024-09-30

**Authors:** Thomas Anklam, René Tannert

**Affiliations:** ^1^ Institute for Materials Research German Aerospace Center (DLR) Linder Höhe 51147 Cologne Germany; ^2^ Institute of Inorganic Chemistry University of Cologne Greinstrasse 4–6 50939 Cologne Germany

**Keywords:** aerogels, hydrophobic, phenolic ethers, resorcinol‐formaldehyde, xerogels

## Abstract

Phenolic aerogels based on resorcinol‐formaldehyde (RF) are among the best thermally insulating materials. However, the hydrophilicity inherent to the free phenolic moiety of RF gels generally limits their actual range of applications. Prior efforts to render phenolic gels hydrophobic are restricted to post‐synthetic functionalizations of hydrophilic gels, processes that are often limited in efficiency, scope, and/or longevity. Here, an acid‐mediated conversion of 1,3,5‐trimethoxybenzene with formaldehyde is reported, yielding monolithic trimethoxybenzene‐formaldehyde (TMBF) aerogels and xerogels with low density (0.11–0.30 g cm^−3^), high porosity (74–92 %), inner surface areas (*S*_BET_) of up to 284 m^2^ g^−1^, and thermal conductivity of 34.5–43.9 mW m^−1^ K^−1^. For a monolithic xerogel based on TMBF xerogels an unprecedently low thermal conductivity of 34.5 mW m^−1^ K^−1^ could be achieved. In addition, all TMBF gels are thermally stable (degradation >280‐310 °C) and highly hydrophobic (water contact angles 130°–156°). As such, TMBF serves as a new class of inherently hydrophobic aerogels and xerogels and useful complement to RF materials.

## Graphical abstract

Thermally stable and highly hydrophobic monolithic trimethoxybenzene‐formaldehyde aerogels and xerogels could be produced from trimethoxybenzene, formaldehyde, organic solvents, and hydrochloric acid. The synthetic control of structural features (cross‐linking, porosity, particle size, inner surface area) and resulting properties (thermal conductivity, stiffness) of the gels are described.

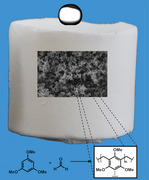

## 1 Introduction

Aerogels are nanostructured open‐porous solids with high porosity that are typically produced by a sol‐gel process followed by drying with supercritical solvent. Their intrinsically low densities and high specific surface areas render them attractive materials for various applications. Inorganic aerogels based on silica, for instance, have been commercialized as insulating materials in both the oil‐and‐gas industry and the building sector.^[^ ^1^ ^]^

Organic aerogels are the subject of research since Pekala's seminal reports on monolithic aerogels of the phenolic thermoset resorcinol‐formaldehyde (RF) with envelope density of (0.04–0.25 g cm^−1^), porosity (>80%), and inner surface area (400–900 m^2^ g^−1^) comparable to silica aerogels.^[^ ^2^ ^]^ Later, it was reported that lightweight RF aerogels can be thermally superinsulating and that RF gels can efficiently be carbonized into electrically conductive carbon aerogels.^[^ ^3^ ^]^ The corresponding phenolic xerogels – denser analogs of aerogels obtained from drying under ambient pressure as opposed to supercritical fluid drying – have also been reported, albeit with somewhat compromised properties, i.e., higher density (0.3–0.6 g cm^−1^), lower porosity (<80%), and lower inner surface (<150 m^2^ g^−1^). The thermal conductivity of monolithic phenolic xerogels has been reported to range from 0.04 to 0.08 W m K^−1^.^[^ ^3^, ^4^ ^]^ However, for a certain granular RF xerogel, a conductivity of 0.035 W m K^−1^ has been reported.^[^ ^4c^ ^]^

In principle, these properties render phenolic aerogels and xerogels promising materials for insulation, filtering, catalysis, and electrochemical energy storage.^[^ ^5^ ^]^ There are, however, certain drawbacks associated with free phenolic aerogels: As is the case for most organic materials, their thermal stability is rather limited. The decomposition of RF xerogels, for instance, has been reported to start well below 200 °C.^[^ ^2^, ^6^ ^]^ Alkylated phenolics, in contrast, are reported to be significantly more stable toward thermal decomposition. Anisole‐formaldehyde resins, for instance, have been reported to withstand temperatures of up to 390 °C.^[^ ^7^ ^]^

**Scheme 1:**
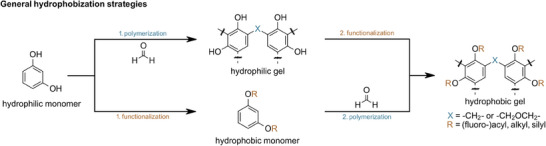
Alternative strategies toward hydrophobic phenolic gels: post‐synthetic functionalization of free phenolic gels (upper path) and direct polymerization of inherently hydrophobic monomers (lower path).

Regarding the wettability of phenolics, the free hydroxy groups of the polymer render the material rather hydrophilic, thus limiting the scope of potential applications to some extent: the uptake of water from air can lead to significant content of liquid water in the aerogel, thereby compromising both sorptive and insulating properties of the gel.^[^ ^8^ ^]^ Furthermore, the integrity and stability of aerogels is at stake due to subsequent evaporation of the liquid adsorbed onto the gel surface – these factors also limit the commercialization of organic aerogels and underline the need for the development of hydrophobic phenolic aerogels and xerogels. A material's wettability can be chemically influenced by manipulation of functional groups on their surface.^[^ ^9^ ^]^ In order to address the hygroscopic behavior of RF aerogels, phenolic hydroxy groups can be functionalized in a post‐synthetic fashion (**Scheme** **1**, upper pathway). Known examples include alkylations of RF xerogels using methanol in the gas phase,^[^ ^10^ ^]^ perfluoroacylation of RF aerogels using perfluoroalkanoic acids,^[^ ^11^ ^]^ and silylations using disilazanes or silyl chlorides.^[^ ^12^ ^]^ Our group has recently come up with a method to silylate RF xerogels using sterically encumbered silyl chlorides and triflates along with amine bases in solution resulting in monolithic materials with pronounced hydrophobicity.^[^ ^13^ ^]^ However, modifications of preformed gel networks generally suffer from incomplete functionalization due to limitations in accessibility of pores, and diffusion control leads to poor reaction kinetics, especially for larger monolithic samples. In that respect, an approach that directly produces hydrophobic materials within the polymerization step would be more favorable (Scheme 1, lower pathway).

The aim of this work was to explore a direct synthetic protocol toward thermally stable and hydrophobic monolithic aerogels starting from phenolic ethers and formaldehyde.

## 2 Results and Discussion

### 2.1 Synthetic Planning

The synthesis of phenolic aerogels and xerogels relies on the polycondensation of phenols to aldehydes. In particular, resorcinol (or phloroglucinol) and formaldehyde are widely used due to their reactivity and the fact that pore sizes can be fine‐tuned from nanometer to micrometer range.^[^ ^3^, ^14^ ^]^ Phenolics are generally produced by means of an electrophilic aromatic substitution reaction between a phenolic derivative and an aldehyde and a subsequent condensation polymerization reaction between the resulting phenolic methylol compounds.^[^ ^15^ ^]^

**Scheme 2:**
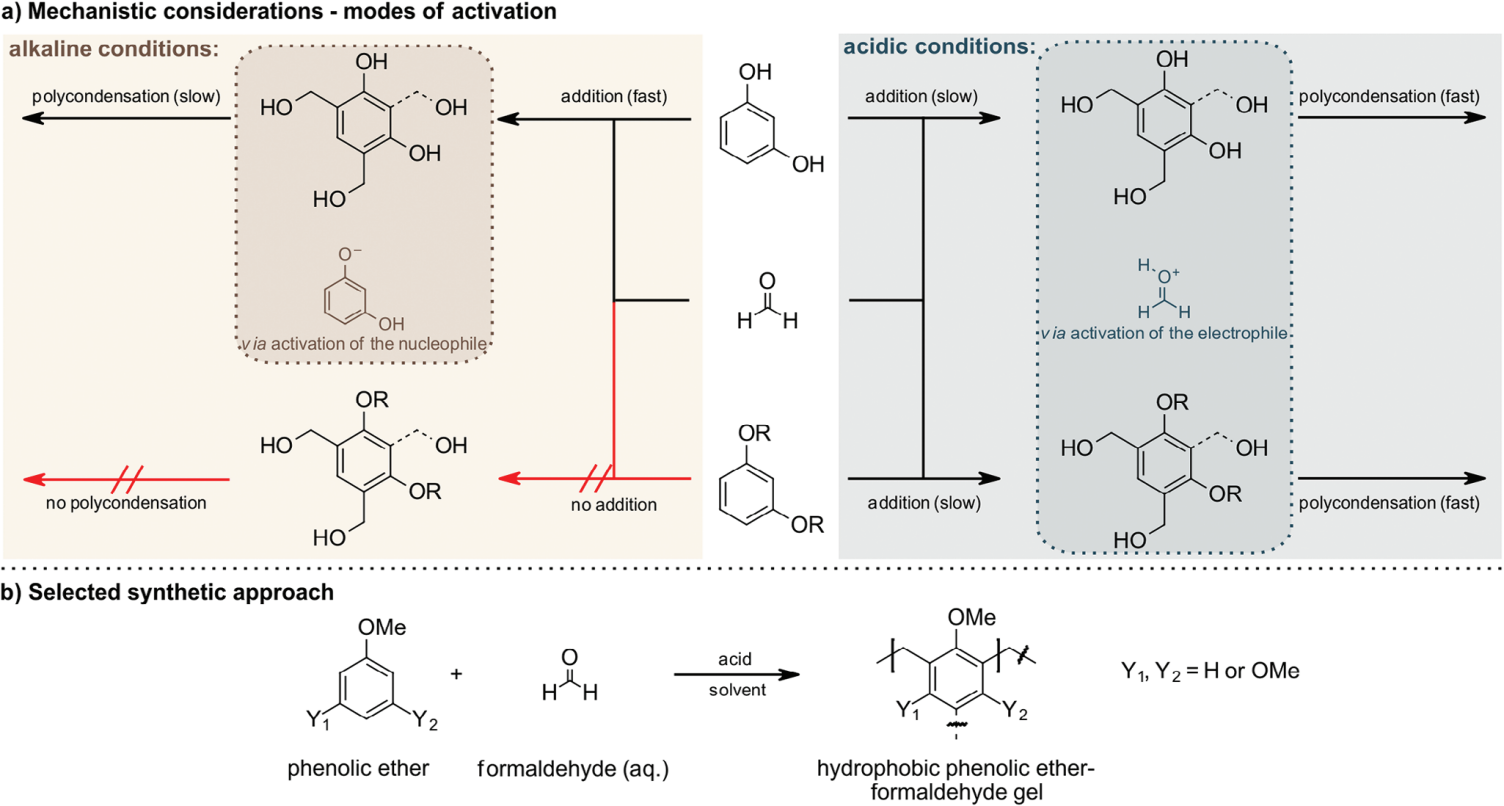
a) The role of the pH value in the addition of formaldehyde to free and alkylated phenols. b) The selected synthetic approach: acid‐promoted additions of formaldehyde to phenolic ethers.

So far, phenolic aerogels are predominantly synthesized using alkaline conditions (**Scheme** **2**a, left part) and employing an excess of formaldehyde (2‐3 equivalents with respect to the phenol employed) in analogy to the industrially relevant Resol‐route^[^ ^16^ ^]^ toward phenolic resins.

Besides relying on base catalysis, one can alternatively apply acidic conditions (Scheme 2a, right part) in analogy to the industrial Novolak‐route.^[^ ^17^ ^]^ These aerogels can be more readily obtained since gelation is generally faster at low pH.^[^ ^6^, ^18^ ^]^ So far, thermal properties of monolithic aerogels made from acidic media have not been demonstrated to reach levels of Pekala‐type aerogels. However, the Leventis group reported on the HCl‐catalyzed synthesis of RF aerogels in acetonitrile that resulted in aerogels with envelope densities as low as 0.1 g cm^−3^, specific surface areas of >500 m^2^ g^−1^ and a microstructure similar to base‐catalyzed RF aerogels synthesized in water.^[^ ^18d^ ^]^ When a direct polymerization of *O*‐functionalized phenols is considered, the mechanism of its addition to formaldehyde has to be taken into account: In alkaline media, deprotonation of the phenol leaves a resonance‐stabilized phenolate that readily adds to unactivated formaldehyde forming a benzylic alcohol derivative.^[^ ^19^, ^20^ ^]^ A drastic example of such an anionic acceleration is provided by oxy‐Cope rearrangements which can be sped up by several orders of magnitude when deprotonated alcohols are used, see refs. [20, 21, 22] Since phenols bearing *O*‐functionalization lack the possibility to form this activated anionic nucleophile, they should be significantly less reactive in said addition to formaldehyde (Scheme 2a, left part). And indeed, Mayr's nucleophilicity parameters for phenolate (N = 18.5 in MeCN)^[^ ^21^ ^]^ and anisole (N = −1.2 in CH_2_Cl_2_)^[^ ^22^ ^]^ differ largely which suggests huge differences in their reaction rates. The sol‐gel polymerization of phloroglucinol and resorcinol with various aldehydes in non‐aqueous solvents had been patented. We were, however, unable to reproduce the synthetic protocols reported therein for ethers. ^[^ ^23^ ^]^

In acidic media, however, it is rather the electrophilic (Friedel‐Crafts‐type) activation through protonation of formaldehyde at low pH that renders the process feasible whereas no further activation of the phenol is to be expected.^[^ ^22^, ^24^ ^]^ As a consequence, the employment of *O*‐functionalized phenols including ethers should be feasible (Scheme 2a, right part). Furthermore, electron‐donating substituents should be beneficial as they increase the nucleophilicity of the arene. Accordingly, the nucleophilicity parameter N is reported 3–4 orders of magnitude higher for dimethoxybenzene (DMB) when compared to anisole, and further increase in nucleophilicity can be expected for 1,3,5‐trimethoxybenzene (TMB).^[^ ^22^, ^25^ ^]^ While there has been no report on aerogels produced from alkyl aryl ethers, some evidence has been provided for analogous polymeric resins made by an acid‐promoted condensation of anisole, DMB, or TMB with formaldehyde. In that context, it was also found that both polymer topology and degree of cross‐linking highly depend on the organic solvent employed: hyperbranched polymers were predominantly achieved using chloroform while the use of tetrahydrofuran resulted in linear polymers.^[^ ^26^ ^]^ However, certain combinations of (halogenated) solvents and acids are also known to result in the selective formation of calixarenes.^[^ ^27^ ^]^

Thus, mechanistic considerations and literature precedence suggested that alkoxyarenes and formaldehyde could deliver gels under acidic conditions and in a solvent‐dependent manner (Scheme 2b).

### 2.2 Synthesis

Commercially available *O*‐methylated analogs of phenol, resorcinol, and phloroglucinol (i.e., anisole, dimethoxybenzene, and trimethoxybenzene) were selected for initial studies. In analogy to RF gel synthesis, a two‐fold excess of formaldehyde was chosen in order to promote sufficient cross‐linking for the formation of gels. As hydrochloric acid had been successfully employed in acid‐mediated formation of RF gels,^[^ ^6^, ^18^ ^]^ it was chosen as promoter.

Even under rather harsh conditions no gel formed using anisole as starting material (Table S1 and Figure S1a, Supporting Information). Work‐up of the reaction mixture after 7 days followed by ^1^H‐ and ^13^C‐NMR spectroscopy revealed almost exclusively unconverted anisole emphasizing its lack of reactivity (Figures S2 and S3, Supporting Information). In contrast, 1,3‐dimethoxybenzene was converted under these conditions, albeit only to resinous precipitates. Such precipitates were also formed at lower concentration of ethers, using less acid, or at lower reaction temperatures (Table S1 and Figure S1b, Supporting Information). The pronounced insolubility of the precipitate in a wide range of solvents (water, EtOH, MeOH, DMSO, MeCN, CHCl_3_, CH_2_Cl_2_, EtOAc, cyclohexane, *n*‐hexane) suggests the formation of polymeric structures, but also prevented more detailed analysis of the resins, e.g., by NMR spectroscopy.

We then turned our attention to TMB, and, to our delight, were able to produce monolithic gels at a variety of synthetic conditions using hydrochloric acid: While the first successful processes involved the treatment of a TMB solution in DMSO (c = 0.6 mol L^−1^) with two equivalents of formaldehyde at 60 °C for a period of 7 days (**Table** **1**, A1), we found that increasing the temperature to 80 °C (Table 1, A2), decreasing the relative amount of HCl to 1.5 mol% (Table 1, A3) and decreasing the concentration of TMB to 0.45 mol L^−1^ (Table 1, A4) also led to the formation of gels in DMSO. The scope of solvents successfully used for gelation furthermore includes 1,4‐dioxane (Table 1, A5), MeCN (Table 1, A6), and AcOH (Table 1, A7), while employment of *N,N*‐dimethylformamide, *N*‐methyl pyrrolidinone and EtOH did not lead to the formation of gels. In additional control experiments performed in the absence of acid or using triethylamine as alkaline catalyst, no gel formation was observed emphasizing the need for an acidic activation as proposed above (Table S1, Supporting Information).

**Table 1:** Conditions for the synthesis of TMBF aerogels (A1–A7) or xerogels (X7 and X8). The formaldehyde/TMB molar ratio was kept constant at 2. Samples were gelled and aged at the respective temperature in an oven for 7 days. For a more detailed summary, see Table S2 (Supporting Information).

Sample	Solvent	c(TMB) [mol L^−1^]	HCl/TMB [mol%]	Temperature [°C]
A1	DMSO	0.60	12.5	60
A2	DMSO	0.60	12.5	80
A3	DMSO	0.60	1.5	80
A4	DMSO	0.45	12.5	80
A5	Dioxane	0.60	12.5	60
A6	MeCN	0.60	12.5	60
A7	AcOH	0.60	12.5	60
X7	AcOH	0.60	12.5	60
X8	AcOH	0.60	12.5	80

With respect to the phenolic reagents employed, TMB is regarded harmful upon swallowing.^[^ ^28^ ^]^ However, resorcinol (hitherto used for the production of most phenolic gels) causes more harm as it is more corrosive, can cause damage to both blood and the central nervous system, and is harmful to aquatic life.^[^ ^29^ ^]^ Therefore, TMB can be regarded less problematic with respect to occupational and environmental safety.

High‐performance liquid chromatography (HPLC) measurements of early stages of the process (until 120 min) revealed that TMB was fully consumed after 0.5 min in MeCN, whereas low amounts of TMB were still detected after 120 min in DMSO, another dipolar‐aprotic solvent. In non‐polar dioxane, on the other hand, TMB is fully consumed after 10 min. Here, the absence of accumulated methylol species^[^ ^30^ ^]^ in HPLC chromatograms indicate that the addition reaction is the rate‐determining step of the polymerization (Figures S4–S6, Supporting Information).

**Figure 1:**
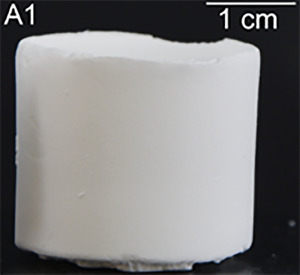
Representative photograph of TMBF aerogel monolith A1.

With the variety of possible synthetic conditions in hand, we envisioned to further extend the scope of this recipe to xerogels by applying ambient pressure drying rather than supercritical drying. The first monolithic xerogel was obtained by using the same synthetic conditions as for sample A7 (Table 1, X7), albeit with cracks throughout the monolith. Increasing the reaction temperature to 80 °C then finally led to intact monolithic xerogels (Table 1, X8). Gelation times ranged between a few hours and several days. In DMSO, an increase in reaction temperature, concentration of arene, and/or ratio of acid resulted in faster gelation. However, the solvent itself also influenced the gelation times, significantly (≈5–10 min in the case of AcOH and MeCN). Irrespective of the applied synthetic conditions, the gels were white in color (**Figure** **1**; Figure S8, Supporting Information).

### 2.3 Structural Characterization

**Figure 2:**
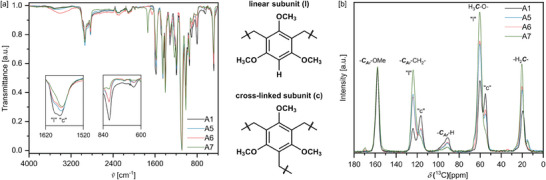
a) ATR‐IR spectra of TMBF aerogels. The spectra were normalized to the band at 1090 cm^−1^. For clarification purposes, magnified regions ≈1570 and 720 cm^−1^ are shown in inserts. b) Solid state ^13^C‐CP‐MAS‐NMR spectra of TMBF aerogels. The spectra were normalized to the signal at 158 ppm.

In order to investigate the chemical structure, attenuated total‐reflection infrared spectroscopy (ATR‐IR) was performed (**Figure** **2**a; Figures S9–S17, Supporting Information). When the resulting spectra were normalized to the characteristic strong band at 1090 cm^−1^, corresponding to C─O stretching vibrations of the methoxy groups, the resulting relative band intensities of other characteristic bands revealed differences for individual gels. More specifically, the intensities of bands at 801 and 1595 cm^−1^ (Figure 2a, denoted l) were found to consistently follow the order A1>A5≈A6>A7. While the former band is associated with out‐of‐plane deformation vibration of aromatic C‐H bonds, the latter band corresponds to aromatic C─C stretching vibrations of partially unsubstituted aromatic carbon‐carbon bonds. These findings suggest that crosslinking is highest in gels made in AcOH (A7) followed by dioxane (A5) and MeCN (A6), and lowest in DMSO (A1).

As a side note, A6 and A7 exhibit absorption bands at 1660 and 1740 cm^−1^, respectively, typical for the stretching vibration of carbonyls. As MeCN (A6) and AcOH (A7) were used as solvents for gelation, amide formation via a Ritter‐type reaction of methylol groups in presence of MeCN and HCl^[^ ^31^ ^]^ or acetylation of methylol groups in presence of AcOH and HCl seems reasonable.

In order to further corroborate the chemical structure of the gels, ^13^C CP‐MAS NMR spectroscopy was conducted (Figure 2b; Figures S18–S21, Supporting Information). All gels share the main peak pattern comprising aromatic carbons adjacent to oxygen at 160 ppm. Aromatic carbon atoms neighboring methylene units appear at 110–130 ppm, while aromatic CH moieties are visible at 85–100 ppm. Peaks at 50–70 ppm are associated with methoxy carbons, and methylene bridges show up at ≈20 ppm.^[^ ^32^ ^]^

The relative intensity of aromatic CH signals versus methylene CH_2_ varies across the different solvents employed in the synthesis: gels made in acetic acid (A7) show only traces of aromatic protons resembling unreacted positions, and, correspondingly the highest relative intensity of methylene signals indicating functionalization of TMB on all three reactive sites. On the other hand, gels made in DMSO (such as A1) retained a significant portion of aromatic CH groups suggesting that these gels are cross‐linked to a lower degree. In addition, minute peaks at 170 ppm in the spectra of A6 and A7 confirm the presence of acyl groups suggested by IR studies.

Throughout gelation, aging, solvent exchange, and drying, the specimens are subject to a certain degree of shrinkage (**Table** **2** and Table S3, Supporting Information). While shrinkage generally only occurred during solvent exchange and drying, samples in dioxane shrunk significantly during aging. The envelope densities of aerogel samples synthesized in DMSO ranged from <0.11 g cm^−3^ to <0.16 g cm^−3^ (Table 2, A1–A4) with sample A4 having the lowest density due to the lower initial concentration of monomers. These findings are in line with studies on acid‐catalyzed RF gels, where slightly higher shrinkage and densities were reported.^[^ ^18d^ ^]^ For aerogels prepared from other solvents, envelope densities ranged between 0.13 and 0.30 g cm^−3^ (Table 2, A5–A7). Changing the drying procedure for sample A7 from supercritical‐ to ambient pressure‐drying increased the envelope density to 0.34 g cm^−3^ (Table 2, X7). When 80 °C was used for gelation instead of 60 °C, the resulting envelope density was 0.1982 g cm^−3^ (Table 2, X8). With respect to organic xerogels, this density is remarkably low.

**Table 2:** Envelope density *ρ*_e_, (radial) shrinkage, porosity *Φ*, and BET surface area *S*_BET_ (derived from N_2_ physisorption) of TMBF xerogels and aerogels.

Sample	Shrinkage [%]	*ρ*_e_ [g cm^−3^]	*Φ* [%]	*S*_BET_ [m^2^ g^−1^]	*V*_p,total_ [cm^3^ g^−1^]	*V*_p,BJH_ [cm^3^ g^−1^]	Contact angle^a)^ [°]
A1	15	0.1423 ± 0.0010	89	0.78^a)^	6.29	‐^b)^	130 ± 4
A2	10	0.1386 ± 0.0004	89	13.3	6.43	0.0332	145 ± 3
A3	16	0.1555 ± 0.0015	88	23.4	5.68	0.0717	147 ± 4
A4	11	0.1083 ± 0.0006	92	38.6	8.51	0.136	156 ± 2
A5	30	0.2991 ± 0.0020	77	284	2.56	1.86	151 ± 3
A6	6.2	0.1254 ± 0.0006	91	2.26^a)^	7.25	‐^b)^	‐^d)^
A7	13	0.1595 ± 0.0034	88	191	5.49	0.418^c)^	153 ± 2
X7	32	0.3371 ± 0.0043	74	180	2.20	0.430^c)^	144 ± 3
X8	18	0.1982 ± 0.0009	85	152	4.29	0.353^c)^	150 ± 4

^a)^ Kr sorption had to be used due to low *S*_BET_;

^b)^ BJH method is not applicable for Kr‐measurements;

^c)^ Hysteresis loop did not close;

^d^ ^)^Droplet was absorbed.

Throughout all samples, apart from the monomer concentration, the overall shrinkage (i.e., changes in diameter between the gelled sample with diameters of the reaction mold and the dried gel monolith) of the materials influenced the envelope density most significantly. As the skeletal densities were generally comparable for all samples (Table S4, Supporting Information), the differences in envelope density resulted in porosities ranging between 74% and 92% (Table 2, Equation S1, Supporting Information). By lowering the initial concentration, stable monolithic aerogels with envelope densities down to 0.11 g cm^−3^ could be obtained. Notably, sample X8 exhibits a porosity of 85%. While such porosity is common for aerogels, it is uncommonly high for xerogels where the porosity typically does not exceed 80%.^[^ ^12^, ^13^, ^33^ ^]^

**Figure 3:**
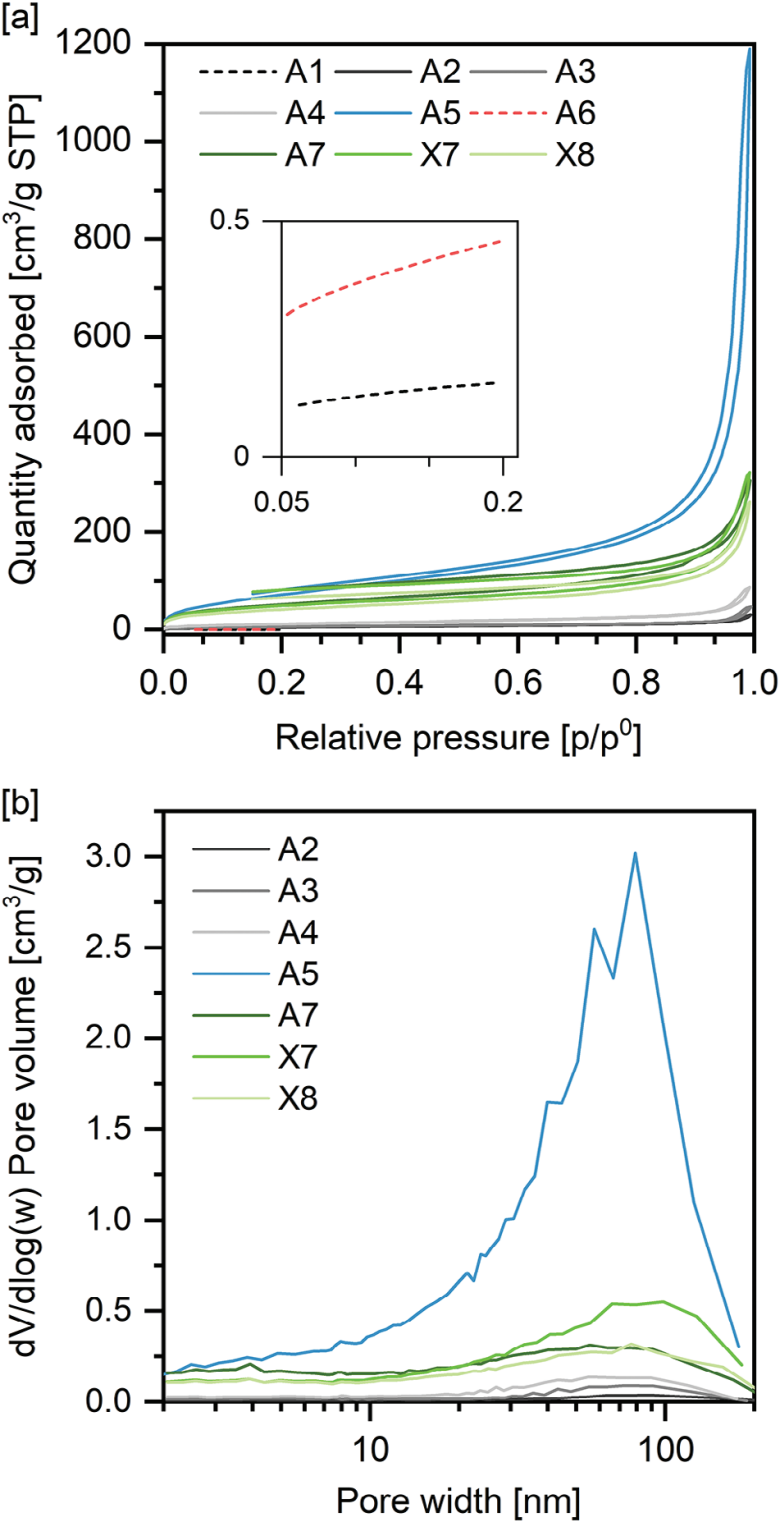
a) N_2_‐sorption isotherms obtained at 77 K (Inset: Kr‐Sorption isotherms). b) BJH pore size distributions derived from the desorption branches of the N_2_‐sorption isotherms.

Surface‐ and pore characteristics of the aero‐ and xerogels were further investigated by means of N_2_‐ and Kr‐ physisorption (**Figure** **3**). With respect to the resulting sorption isotherms, the degressive increase in adsorbed quantity at low relative pressures of nitrogen, as well as the narrow (H3‐type) hysteresis are reminiscent of type IV(a) isotherms. The occurrence of a low‐pressure hysteresis, in particular in samples made in AcOH, indicates some deformation of the network upon entry of the adsorbate molecules into pores.^[^ ^34^, ^35^ ^]^ However, the progressive increase in adsorbed quantity at very high relative pressures (absence of a plateau) is more reminiscent of type II‐ isotherms.^[^ ^36^ ^]^ Analyses of the inner surface area using the method of Brunauer, Emmett, and Teller (BET)^[^ ^37^ ^]^ revealed a wide range of surface areas, depending on the synthetic conditions. While the dipolar‐aprotic solvents acetonitrile and DMSO yielded materials with low surface areas (≈2 m^2^ g^−1^; Table 2, A1 and A6), the less dipolar dioxane and polar‐protic acetic acid yielded aerogels with surface areas of 284 m^2^ g^−1^ (Table 2, A5) and 191 m^2^ g^−1^ (A7), respectively.

In case of DMSO as solvent, an increase of the surface area was found when the gelation temperature was increased from 60 to 80 °C (Table 2, A1 vs A2). At 80 °C, lower concentrations of either TMB or HCl led to higher inner surface areas (Table 2, A2–A4). The influence of the drying method on the resulting surface was studied on samples A7 and X7 representing aerogel and xerogel, respectively. As expected, the higher shrinkage for xerogel X7 was accompanied by a larger decrease in inner surface area when compared to aerogel A7. In case of X7, however, an impressive inner surface area of 180 m^2^ g^−1^ remained after drying at ambient pressure.

Synthesizing gels in acetic acid at 80 °C instead of 60 °C improved the shrinkage during drying, but lowered the surface area slightly to 150 m^2^ g^−1^ (Table 2, X7 vs X8).

Pore size distributions were extracted from the desorption branch of the isotherm according to the method of Barret, Joyner, and Halenda (BJH).^[^ ^38^ ^]^ Pore sizes ranged from 2 to 200 nm covered by the BJH method, with a maximum of 50–90 nm.

For aerogels made in DMSO, the BJH pore volume increased at elevated gelation temperature and by further decreasing TMB concentration or HCl/TMB ratio (Table 2, A2–A4). While samples synthesized in AcOH exhibited moderate BJH pore volumes (Table 2, A7 and X7–X8) for gels synthesized in dioxane a significantly higher BJH pore volume was determined (Table 2, A5). The pore volume as determined using the BJH method correlated with surface areas derived from BET analysis (Figure 3b). The total pore volume was calculated from envelope and skeletal densities as determined by pycnometry (Equation S2, Supporting Information). For all samples, total pore volume exceeded mesopore volumes derived from BJH significantly. The effect of the drying procedure was investigated on samples A7 (supercritical drying) and X7 (ambient‐pressure drying). For xerogel X7, a significantly lower total pore volume was determined when compared to its analog A7 which was dried in a supercritical fluid. Interestingly, their BJH pore volume was in a similar range. This outcome indicates that the evaporation of pore liquid at ambient pressure mainly led to the destruction of macropores rather than mesopores. Such a result is unexpected since upon evaporation of solvent the increased capillary pressures in smaller pores should collapse them first.^[^ ^39^ ^]^

Overall, the physisorption experiments indicate predominantly macroporous without significant microporosity. Depending on the solvent employed in gelation, a degree of mesoporosity can be selectively added, albeit with a rather broad distribution of porosity.

**Figure 4:**
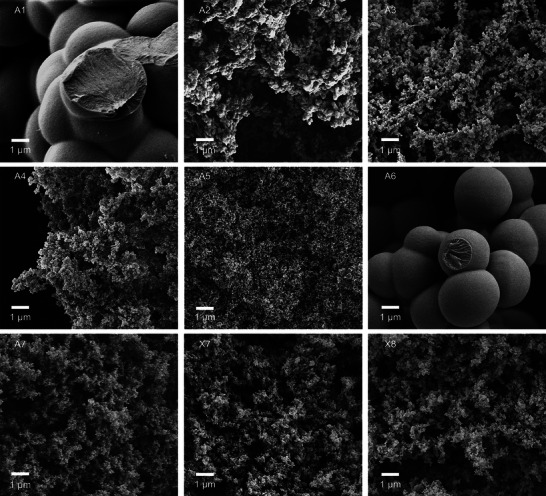
SEM images of aerogel and xerogels.

Scanning electron microscopy (SEM) corroborated the presence of macropores in all TMBF samples (**Figure** **4**; Figures S23–S31, Supporting Information). While most samples consisted of macropores in the µm regime, sample A5 made from dioxane comprised significantly smaller macropores. The gels’ microstructures were generally composed of conjoined spherical particles. As expected for such materials, samples with larger BET surface area comprise smaller particles.

Both the degree of interconnection and size of microparticles varied significantly depending on the synthetic conditions of the material: In samples from dipolar‐aprotic solvents DMSO and MeCN, the biggest microparticles could be determined (Figure 4, A1 and A6), while the material with smallest microparticles was obtained in nonpolar dioxane (A5). In DMSO, an increase of the synthesis temperature from 60 to 80 °C resulted in significantly smaller microparticles (Figure 4, A1 vs A2). A further decrease of particle size could be achieved by either decreasing the ratio of acid‐to‐TMB (A2 vs A3) or by reducing the absolute concentration of the TMB sol (A2 vs A3). Samples synthesized in AcOH consisted of microparticles with sizes in between those of dipolar‐aprotic and nonpolar solvents (Figure 4, A7, X7, and X8).

Whereas the microstructure cannot be correlated to the kinetics of the early stages of the polymerization, there is a pronounced effect of the solvent's polarity indicating that thermodynamic factors such as solubility govern the formation of the porous polymer network. Similar findings have been reported for base‐catalyzed reactions of resorcinol and formaldehyde.^[^ ^19b^ ^]^

In summary, the microstructures of TMBF can be conveniently tailored by the choice of synthetic parameters similar to what was found for analogous RF formulations.

### 2.4 Functional Characterization

In order to assess the wetting behavior of the TMBF aerogels and xerogels, contact angles of water droplets on the gel monoliths were determined (Table 2). In contrast to RF aerogels, all TMBF samples displayed non‐wetting behavior with contact angles ranging from 130° ± 4° to 156° ± 2°, rendering the materials (super‐)hydrophobic (Figure S32, Table S5, and Videos S1–S4, Supporting Information). Although hydrophobic, sample A6 slowly absorbed the droplets entirely, a phenomenon that may be explained by its combination of pronounced macroporosity and large particles. As a general notion, contact angles were larger for samples with small primary particles and a higher specific surface area.

**Figure 5:**
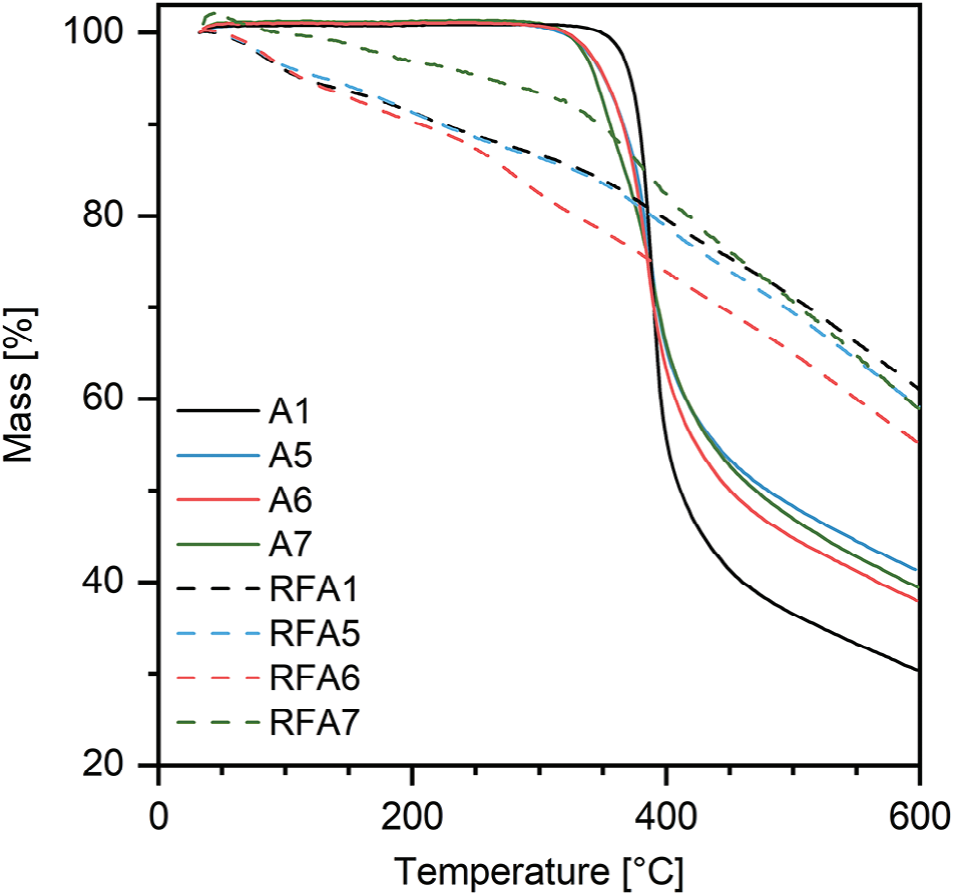
Thermogravimetric analysis of TMBF aerogels.

Thermogravimetric analysis (TGA) was performed in order to investigate the thermal stability of TMBF aerogels (**Figure** **5**, A1, A5–A7). First, the absence of any mass loss at low temperatures that would indicate physisorbed water underlines the hydrophobic nature of all TMBF gels investigated. Second, thermal decomposition was induced at temperatures of 280–310 °C depending on the gel formulation. Heating of the samples to 600 °C was accompanied by mass losses of 59–70%. For comparison, a subset of free phenolic analogs of TMBF was prepared from resorcinol and formaldehyde using the same synthetic conditions and subjected to TGA (Figure 5, RFA1, RFA5, RFA6, RFA7). These gels not only revealed their hydrophilic nature by releasing physisorbed water below 100 °C but, more importantly, also degraded much more readily when heated further indicating the key influence of phenolic methyl groups on the thermal stability of the parent gels. Indeed, for free phenolic resins mass losses at moderate temperatures (<300 °C) were reported due to formation of biaryl ethers.^[^ ^40^ ^]^ Such a net condensation process is not feasible for alkylated phenols. Above 400 °C, the degradation is more pronounced for TMBF gels than for RF gels, presumably due to the lack of formation of thermostable intermediates. In essence, TMBF is very stable up to 300 °C indicating a better suitability of the material for use at low to moderately high temperatures when compared to RF.

The thermal conductivities of TMBF gels were determined using the transient plane source technique (**Table** **3**; Tables S6–S10, and Figures S33–S37, Supporting Information). Regarding aerogels, the lowest thermal conductivities were determined as 34.5 mW m^−1^ K^−1^ for A7, a value that can be considered moderate for aerogels. However, xerogel sample X8 turned out to perform equally well as thermal insulator. Such a low thermal conductivity has so far not been reported for monolithic organic xerogels.

**Table 3:** Thermal and compressive properties of TMBF xerogels and aerogels.

Sample	Thermal conductivity λ [mW m^−1^ K^−1^]	Nominal compr. modulus *E*_C_ [MPa]	Compr. strength σm [MPa]	Specific stiffness [MPa g^−1^ cm^3^]
A1	40.1 ± 2.4	3.40 ± 0.65	0.262 ± 0.025	23.9
A2	43.9 ± 0.7	3.50 ± 0.72	‐	25.3
A5	43.8 ± 0.6	21.0 ± 4.0	3.74 ± 0.79	70.2
A7	34.5 ± 0.1	4.55 ± 1.00	0.344 ± 0.051	28.6
X7	^a)^	18.9 ± 0.6	2.16 ± 0.01	56.0
X8	34.5 ± 0.2	9.12 ± 1.21	0.734 ± 0.092	46.0

^a)^ Not measured (material was cracked).

A low thermal conductivity can generally be achieved when the material has small mesopores to make use of the *Knudsen* effect and an optimal density, low enough that not too much material, which has a high thermal conductivity, is present and high enough for pores to become small enough. This explains why sample A5, which has the biggest BJH pore volume is still not the best of the measured samples. Due to extensive shrinkage, the density of the material became quite high, and therefore a lot of the solids thermal conductivity contributes to the overall thermal conductivity. A7 and X8 on the other hand have a mediocre BJH pore volume but a rather low envelope density. For A1 and A2, the nearly complete absence of mesopores results in higher values than for sample A7, even though the envelope density is in the same size region.

**Figure 6:**
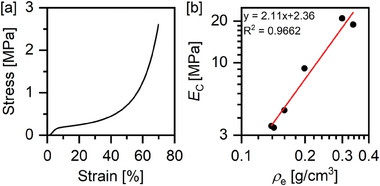
a) Typical stress‐strain curve associated with compressive deformation of TMBF gels (A2). b) Double‐logarithmic plot of stiffness *E*_C_ against envelope density *ρ*_e_. The slope of the regression line represents the scaling exponent *m*.

In order to characterize their mechanical behavior, gels were subjected to quasi‐static uniaxial compression (Tables S11–S16 and Figures S38–S43, Supporting Information). The corresponding stress‐strain plots show different characteristic deformation stages (**Figure** **6**): At low strains (depending on the gels at nominal strains between 2% and 7%), elastic deformation takes place as indicated by the linearity of the stress‐strain curve in that region. The subsequent formation of a stress plateau indicates inelastic deformation caused by bending of cell walls.^[^ ^41^ ^]^ Most of the gels investigated (all but A2) yielded nominal strains between 15% and 33% corresponding to compressive strength ranging between 0.26 and 3.7 MPa. The exponential rise of stress at higher strain levels (>30%) is associated with densification of the material. Such a behavior is typical for aerogels and other cellular materials.^[^ ^42^ ^]^

Depending on their chemical formulations, the TMBF gels did show some variation in terms of stiffness with compressive moduli between 3 and 21 MPa with the highest values achieved using either dioxane or AcOH. The corresponding specific moduli were as high as 70 MPa g^−1^ cm^3^ (Table 3).^[^ ^43^ ^]^ Thus, TMBF seems as variable in terms of balancing flexibility and stiffness as is known for RF and other free phenolic aerogels and xerogels.^[^ ^2^, ^44^ ^]^

For cellular materials, Gibson and Ashby described a power‐law relationship between compressive modulus *E* and envelope density *ρ*_e_ according to

(1) $$E\propto \rho_{e}^{m}$$

with exponent *m* being calculated as 2.0 for perfectly regular open‐cell foams.^[^ ^45^ ^]^ For a variety of Pekala‐type aerogels, scaling exponents between 2.5 and 2.9 were determined.^[^ ^44^ ^]^ Taking all TMBF gels presented herein into consideration, a scaling exponent of 2.1 results (Figure 6). Thus, TMBF gels seem to consist of a significantly more regular cell structure than Pekala‐type RF.

## 3 Conclusion

In summary, we have established an acid‐catalyzed synthesis of TMBF aerogels and xerogels in organic solvents. The procedure involves the use of commercially available reagents and solvents that can be used without further drying/purification. In addition, trimethoxybenzene is considerably less harmful than resorcinol which is typically used as starting material for phenolic aerogels. Gelation occurs at room temperature within minutes, and subsequent aging and drying readily yield the corresponding aerogel or xerogel, respectively. The porous structure can be tuned by variation of the synthesis temperature, the concentrations of starting materials and acid, and especially the choice of solvent. These parameters also influence the resulting mechanical properties, as demonstrated by varying stiffness of the gels, albeit envelope density plays the dominant role in this regard. Microstructure and, consequently, thermally insulating and mechanical properties of the resulting gels could be synthetically adjusted in a manner similar to RF aerogels and xerogels. In contrast to RF, however, TMBF is highly hydrophobic and, thermally considerably more stable.

Gels made in DMSO gave the lowest envelope density/ highest porosity (92%), and were thermally most stable. Very stiff aerogels with the highest mesopore area within this series of aerogels (284 m^2^ g^−1^) could be obtained in 1,4‐dioxane. Of all solvents, the use of MeCN resulted in aerogels with the lowest shrinkage (6%), along with high porosity (91%). Finally, gels produced in AcOH turned out to be the best thermal insulators: irrespective of the drying method applied, both aerogels and xerogels achieved a thermal conductivity of 34.5 mW m^−1^ K^−1^ which, while moderate for the aerogel is excellent for xerogels.

More detailed studies toward understanding of the influence of solvents on structure and properties of the resulting gels are ongoing and will be reported in due course.

Overall, TMBF represents a useful new class of phenolic aerogels that overcomes some of the limitations of the classical RF systems. In future, TMBF may thus serve as a functional material for a variety of applications, e.g., as moisture‐tolerant lightweight‐insulation material.

## Conflict of Interest

The authors declare no conflict of interest.

## Supporting information



Supporting Information

Supplemental Video 1

Supplemental Video 2

Supplemental Video 3

Supplemental Video 4

## Data Availability

The data that support the findings of this study are available from the corresponding author upon reasonable request.
