# Supplementary material for: Hydrophobic Aerogels and Xerogels based on Trimethoxybenzene‐Formaldehyde
Source: Macromol Rapid Commun. 2024 Sep 30;46(2):2400691. doi: 10.1002/marc.202400691 (PMC11756868; doi:10.1002/marc.202400691)
Supplement: Supplementary file 1 — Supporting Information [file MARC-46-2400691-s001.docx]

**Supporting Information**

**Hydrophobic Aerogels and Xerogels Based on Trimethoxybenzene-Formaldehyde**

Thomas Anklam^[a,b]^, René Tannert^*[a]^

[a] German Aerospace Center, Institute for Materials Research, Department of Aerogels and Aerogel Composites, Linder Hoehe, 51147 Cologne, Germany

[b] University of Cologne, Department of Chemistry, Institute of Inorganic Chemistry, Greinstraße 4-6, 50939 Cologne, Germany

* Corresponding author: [rene.tannert@dlr.de](mailto:rene.tannert@dlr.de)

Table of Contents

[Synthesis of aerogels and xerogels 2](#_Toc166860522)

[Chemicals & devices for synthesis 2](#_Toc166860523)

[General procedure for the synthesis of polymeric gels 2](#_Toc166860524)

[Preliminary experiments 3](#_Toc166860525)

[NMR Spectroscopy of an Anisole-Formaldehyde Reaction Mixture 4](#_Toc166860526)

[Reaction monitoring via HPLC 5](#_Toc166860527)

[Drying procedures 8](#_Toc166860528)

[Individual synthetic conditions and resulting gels 9](#_Toc166860529)

[Characterization of aerogels and xerogels 10](#_Toc166860530)

[ATR-IR Spectroscopy 10](#_Toc166860531)

[^13^C CP-MAS NMR 15](#_Toc166860532)

[Determination of shrinkage 17](#_Toc166860533)

[Pycnometry and determination of porosity and total pore volume 18](#_Toc166860534)

[Physisorption 19](#_Toc166860535)

[Scanning electron microscopy (SEM) 19](#_Toc166860536)

[Wetting Behavior 29](#_Toc166860537)

[Thermal Stability 30](#_Toc166860538)

[Thermal conductivity 30](#_Toc166860539)

[Mechanical properties - Compression tests 34](#_Toc166860540)

[References 38](#_Toc166860541)

# Synthesis of aerogels and xerogels

## Chemicals & devices for synthesis

Anisole (99%, Carl Roth), 1,3-dimethoxybenzene, (98%, Alfa Aesar), 1,3,5-trimethoxybenzene (99%, ACR16055 from Acros Organics/ Thermo Fisher Scientific or Sigma Aldrich), resorcinol (99%, W358908-10KG from Sigma Aldrich), aqueous formaldehyde (37%, stabilized with 10% methanol, Sigma Aldrich), DMSO (>99.8%, ChemSolute), 1,4-dioxane (>99.5%, stabilized with BHT, ChemSolute), acetonitrile (HPLC grade), acetic acid (technical, VWR), ethanol (99%, denatured, Th. Geyer), aq. hydrochloric acid (37% *w*/*w*, PanReac AppliChem) and aq. sulfuric acid (96% *w*/*w*, VWR) were obtained from VWR International GmbH (Darmstadt, Germany) and/ or TH. GEYER GMBH & CO. KG (Renningen, Germany) and were used without further purification. Deionized water was obtained by passing tap water through an Envi Green-Line DIA 6000 MB purification cartridge (EnviroFALK, Westerburg, Germany) was used in all experiments. Gelation was, depending on the volume of the precursor solution, either carried out in cylindrical polypropylene (PP)-containers with an average diameter of 32.37 mm or in 2.0 mL SafeLock PP-containers and liquids were transferred using *Research Plus* piston-pipettes (both from Eppendorf, Wesseling, Germany). For heating of samples, universal ovens Memmert UF110 or UF30, (Memmert GmbH + Co. KG, Schwabach, Germany) or safety drying chamber Binder FDL 115 (BINDER GmBH, Tuttlingen, Germany) were used. Preliminary experiments conducted in a 1mL-scale were heated using an Eppendorf ThermoMixer F2.0 equipped with thermally insulating lid (Th. Geyer GmbH & Co. KG, Lohmar, Germany).

## General procedure for the synthesis of polymeric gels

In a typical procedure, the appropriate amount of TMB was dissolved in the respective volume of solvent. The concentrations of TMB were given as molar amount per L of organic solvent, not taking the volume of acid and formaldehyde into consideration. After complete dissolution, an aqueous solution of formaldehyde (F, 37% *m/m*) was added in one portion followed by mixing the solution by manually shaking the sealed container for 15 s and subsequent addition of an aqueous solution of hydrochloric acid catalyst (H). The solution was immediately mixed by shaking the sealed container for 15 s and placing in an oven. The containers were placed in the middle of the preheated oven, avoiding direct contact with the heating walls, air circulation was set to 100% and the outlet valve was opened by 10%. Gelation and aging were carried out for a period of 7 days. Afterwards, the gels were subjected to a sequence of immersion into ethanol (5x initial volume of sol for a period of at least 6 h), and draining of the solvent (5 iterations).

## Preliminary experiments

In order to assess the feasibility of phenolic reagents to undergo gelation, preliminary gelation experiments were conducted on a scale of 1mL to 10 mL according to the general procedure described above. Individual conditions used for the synthesis of gels are summarized in **Table S1.**

**Table S1:** Summary of the preliminary experiments.

| # | c(Arene) [mol/L] | Solvent | Acid | Acid/TMB [mol-%] | Temp. [°C] | Time [days] | Anisole | DMB | TMB |
| --- | --- | --- | --- | --- | --- | --- | --- | --- | --- |
| 1 | 0.6 | DMSO | HCl | 12.5 | 60 | 7 | Solution | Precipitate | Gel |
| 2 | 0.6 | DMSO | HCl | 12.5 | 80 | 7 | Solution | Precipitate | Gel |
| 3 | 0.6 | DMSO | H_2_SO_4_ | 12.5 | 80 | 7 | Solution | not tested | not tested |
| 4 | 0.6 | DMSO | H_2_SO_4_ | 50.0 | 80 | 7 | not tested | Precipitate | not tested |
| 5 | 1.2 | DMSO | H_2_SO_4_ | 50.0 | 80 | 7 | not tested | Precipitate | not tested |
| 6 | 0.6 | DMSO | HCl | 50.0 | 80 | 7 | Solution | Precipitate | Gel |
| 7 | 0.6 | DMSO | HCl | 50.0 | [a] | [a] | Solution | Precipitate | Gel |
| 8 | 0.6 | Dioxane | HCl | 12.5 | 60 | 7 | Solution | Precipitate | Gel |
| 9 | 0.6 | MeCN | HCl | 12.5 | 60 | 7 | Solution | Precipitate | Gel |
| 10 | 0.6 | MeCN | HCl | 50.0 | 60 | 7 | Solution | Precipitate | Gel |
| 11 | 0.6 | MeCN | H_2_SO_4_ | 50.0 | 60 | 7 | Solution | Precipitate | Gel |
| 12 | 0.6 | MeCN | CH_3_SO_3_H | 50.0 | 60 | 7 | Solution | Precipitate | Gel |
| 13 | 0.6 | MeCN | [b] | [b] | 60 | 7 | not tested | not tested | Solution |
| 14 | 0.6 | MeCN | - | - | 60 | 7 | Solution | Solution | Solution |
| 15 | 0.6 | AcOH | HCl | 12.5 | 60 | 7 | Solution | Precipitate | Gel |
| 16 | 0.6 | DMF | HCl | 12.5 | 60 | 7 | Solution | Solution | Solution |
| 17 | 0.6 | NMP | HCl | 12.5 | 60 | 7 | Solution | Solution | Solution |
| 18 | 0.6 | EtOH | HCl | 12.5 | 60 | 7 | Solution | Solution | Precipitate |

[a]: Samples were left for 1 h at 100 °C followed by 24 h at 80 °C. [b] 50mol-% NEt_3_ was employed


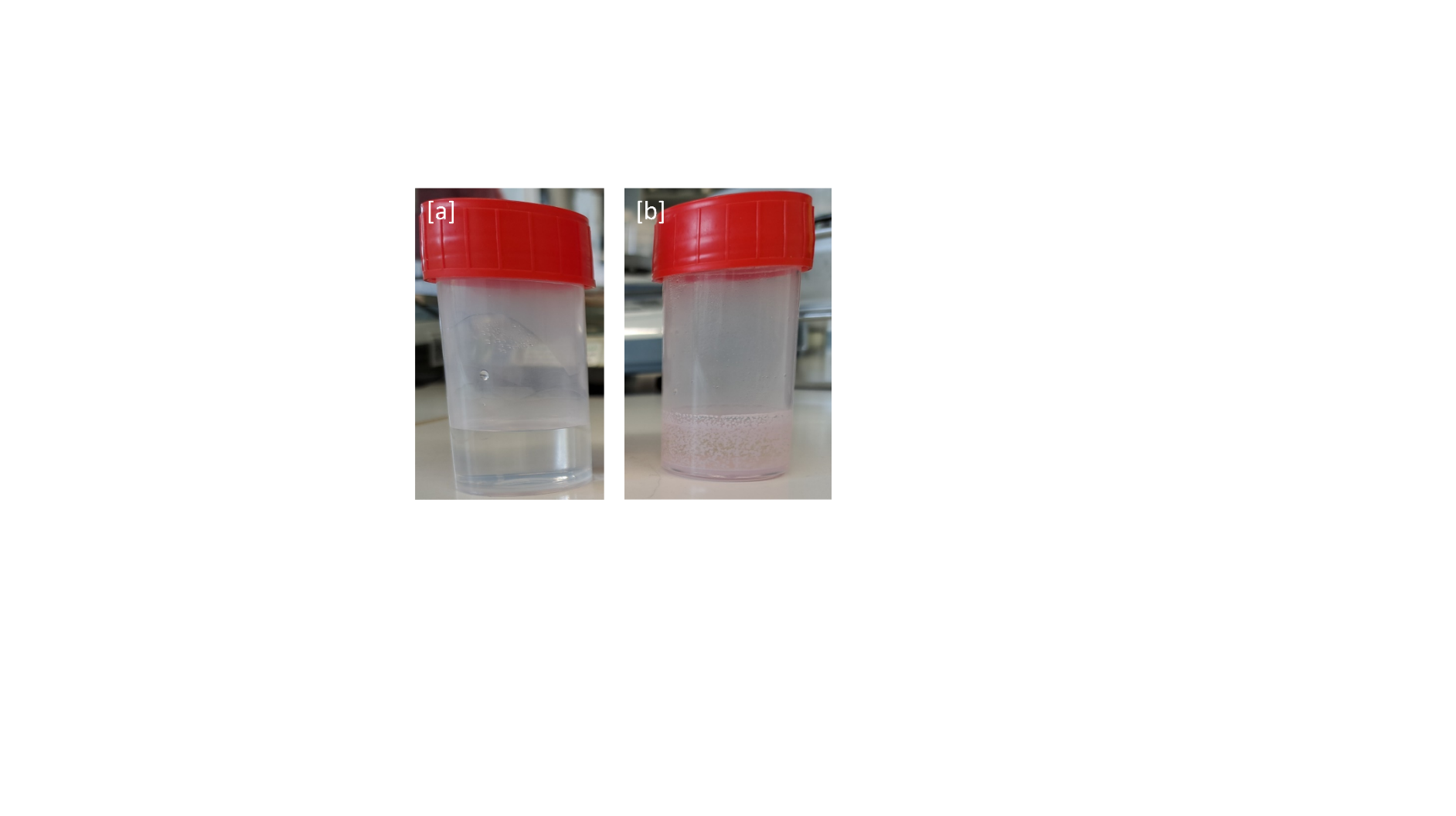


**Figure S1:** Photographs of representative mixtures of formaldehyde and [a] anisole or [b] 1,3-dimethoxybenzene after heating at 80°C for 7 days.

## NMR Spectroscopy of an Anisole-Formaldehyde Reaction Mixture

The reaction mixture from **Table S1**, line 6 comprising anisole, was extracted with ethyl acetate (20mL).The extract was washed with water (3x 10 mL) and dried using magnesium sulfate. The dried organic layer was concentrated at 40°C using a Büchi R-100 rotary evaporator (BÜCHI Labortechnik GmbH, Essen, Germany). The residue was taken up in chloroform and transferred to a NMR tube. Solution phase ^1^H-and ^13^C- Nuclear Magnetic Resonance (NMR) spectroscopy (**Figures S2 and S3)** were conducted on a Spinsolve 80 Ultra spectrometer (Magritek, Aachen, Germany).


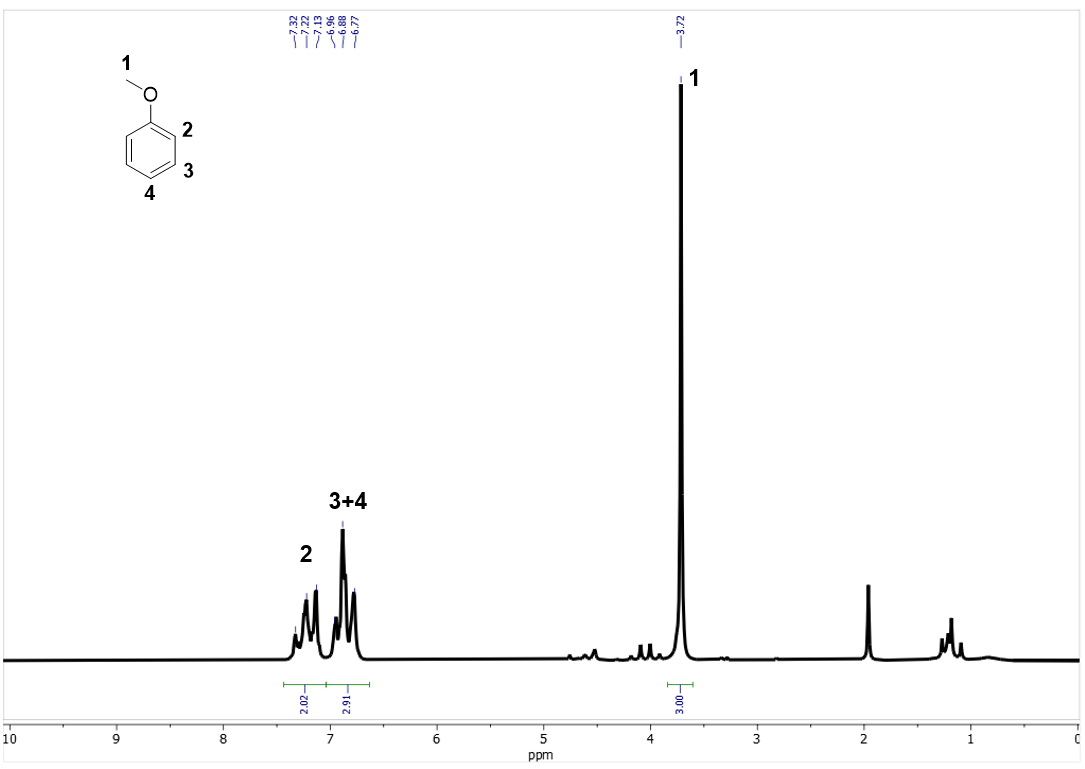


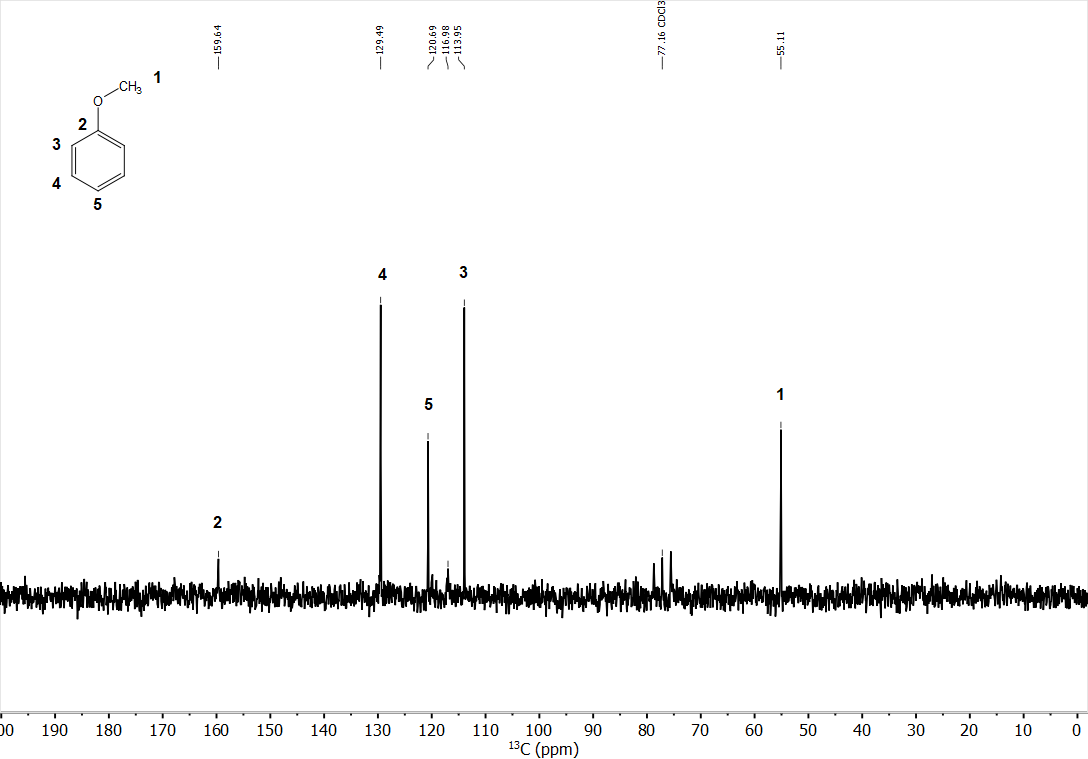
**Figure S2:** ^1^H-NMR spectrum of an anisole formaldehyde reaction mixture.


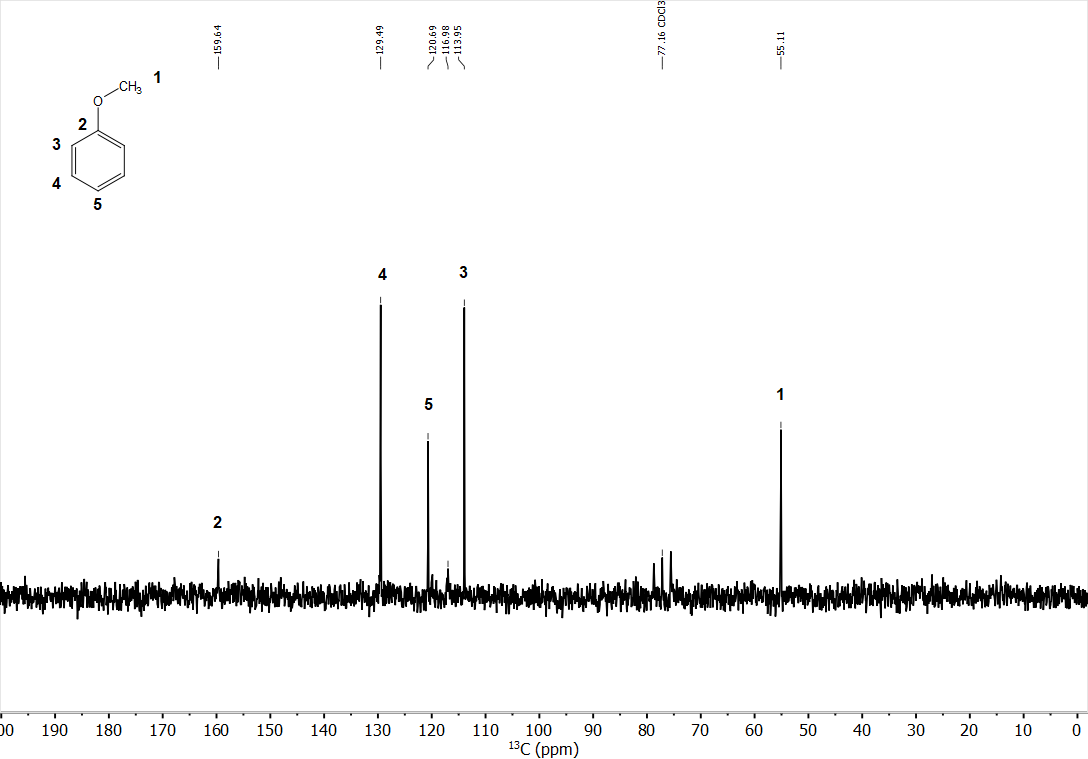


**Figure S3:** ^13^C-NMR spectrum of an anisole formaldehyde reaction mixture.

## Reaction monitoring via HPLC

The consumption of phenolic precursors upon hydroxymethylation and polycondensation was monitored using Reversed Phase-High-Performance Liquid Chromatography (RP-HPLC). For that purpose, a Knauer Azura machine composed of an AS 6.1L autosampler, a P 6.1L quaternary gradient pump, a DAD 2.1L diode array detector (λ = 190 – 700 nm) and a column thermostat was employed (all from KNAUER Wissenschaftliche Geräte GmbH, Berlin, Germany). An Eurospher 100-5 C18 column (length 150 mm, inner diameter 4 mm, particle size 5 μm) was used as stationary phase and acetonitrile (A) and water (B) were used as eluents. Each sample was eluted with A using a linear gradient of B from 30% to 0% within 3 min. Isocratic elution with 100% A continued for 22 min, before a linear gradient of B from 0% to 30% within 1 min was applied. Afterwards, isocratic elution with 70% A and 30% B was continued for 4 min, resulting in a total run time of 30 min. Acquisition, processing and evaluation of data was conducted using the ClarityChrom 8.1 software. The linear relationship between TMB concentration and peak integrals was confirmed by analyzing TMB solutions with concentrations of 0.6 mol/L, 0.3 mol/L, 0.15 mol/L, 0.075 mol/L, 0.0375 mol/L and 0.01875 mol/L at a detector wave length of 245 nm (**Figure S4**). These solutions were prepared by a sequential dilution series starting with a 1.2 mol/L solution of TMB in a 3.6 mmol/L solution of NEt_3_ in MeCN.





**Figure S4:** Confirmation of the linear relationship between TMB concentration and peak integral.

For reaction monitoring, 10 μL aliquots of the reaction solution were diluted in 990 μL of a 3.6 mmol/L solution of NEt_3_ in MeCN to quench the catalyst. The column thermostat was preheated to 40°C before each series of measurements and 0.5 μL of analyte solution was injected for each measurement. Successful quenching of the catalyst was confirmed by analyzing an aliquot that was diluted in a 3.6 mmol/L solution of NEt_3_ in MeCN in one case and in MeCN in the other case, right after dilution and again after 100 min of standing at room temperature (**Figure S5**).





**Figure S5:** Confirmation of catalyst quenching with NEt_3_.

The chromatograms and concentration curves of TMBF reaction mixtures in DMSO, Dioxane, MeCN, AcOH and EtOH are shown in **Figure S6**.





**Figure S6:** HPLC chromatograms of early stages of TMBF reaction mixtures using DMSO (A1), 1,4-Dioxane (A5), MeCN (A6), AcOH (A7) and EtOH as solvents. The respective concentration curves of TMB are shown in the bottom right diagram.

## Drying procedures

For supercritical drying, dip-tube CO_2_ (≥99.5%) obtained from Linde AG (München, Germany) was used. Alcogels were wrapped in tea bags pre-soaked with ethanol and subsequently transferred to a 60 L autoclave vessel (Eurotechnica, Bargteheide, Germany). The vessel was sealed and pressurized to 40-50 bar in order to allow for volume expansion of the ethanol and subsequent draining of pore solvent (solvent spillage effect).^[47]^ Afterwards, the vessel was further pressurized (to 115 bar) and heated to 60°C, and supercritical fluid extraction was performed until ethanol and CO_2_ formed one supercritical phase and, thus ethanol could not be drained from the separator unit anymore. Finally, aerogels were obtained by slowly reducing the pressure in the autoclave to atmospheric pressure at 60°C. The whole drying process took 1-3 days, depending on the loading of the autoclave and the size of the gels. A representative drying protocol is shown in **Figure S7**.





**Figure S7:** Representative protocol for supercritical drying of TMBF gels.

For ambient-pressure drying, the alcogels were taking out of the ethanol bath and directly transferred to PP molds, placed into an oven and left at 60 °C for at least 1 day.

## Individual synthetic conditions and resulting gels

The exact amounts of chemicals used for the synthesis of gels, as well as the drying techniques applied are summarized in **Table S2**:

**Table S2:** Detailed summary of the recipes for the synthesis of relevant TMBF aerogels and xerogels.

| # | Solvent | V(Solvent) [mL] | m(TMB) [g] | c(TMB) [mol/L] | V(F) [mL] | F/Ether  [mol.%] | V(HCl) [mL] | HCl [mol.%] | Temp. [°C] | Drying method |
| --- | --- | --- | --- | --- | --- | --- | --- | --- | --- | --- |
| A1 | DMSO | 20.0 | 2.02 | 0.60 | 1.787 | 200 | 0.124 | 12.5 | 60 | sc |
| A2 | DMSO | 10.0 | 1.01 | 0.60 | 0.8940 | 200 | 0.0620 | 12.5 | 80 | sc |
| A3 | DMSO | 10.0 | 1.01 | 0.60 | 0.8940 | 200 | 0.00800 | 1.50 | 80 | sc |
| A4 | DMSO | 10.0 | 0.760 | 0.45 | 0.6700 | 200 | 0.0470 | 12.5 | 80 | sc |
| A5 | Dioxane | 20.0 | 2.02 | 0.60 | 1.787 | 200 | 0.124 | 12.5 | 60 | sc |
| A6 | MeCN | 20.0 | 2.02 | 0.60 | 1.787 | 200 | 0.124 | 12.5 | 60 | sc |
| A7 | AcOH | 20.0 | 2.02 | 0.60 | 1.787 | 200 | 0.124 | 12.5 | 60 | sc |
| X7 | AcOH | 20.0 | 2.02 | 0.60 | 1.787 | 200 | 0.124 | 12.5 | 60 | apd |
| X8 | AcOH | 20.0 | 2.02 | 0.60 | 1.787 | 200 | 0.124 | 12.5 | 80 | apd |

Gels were obtained as white cylindrical monoliths as shown in **Figure S8**:


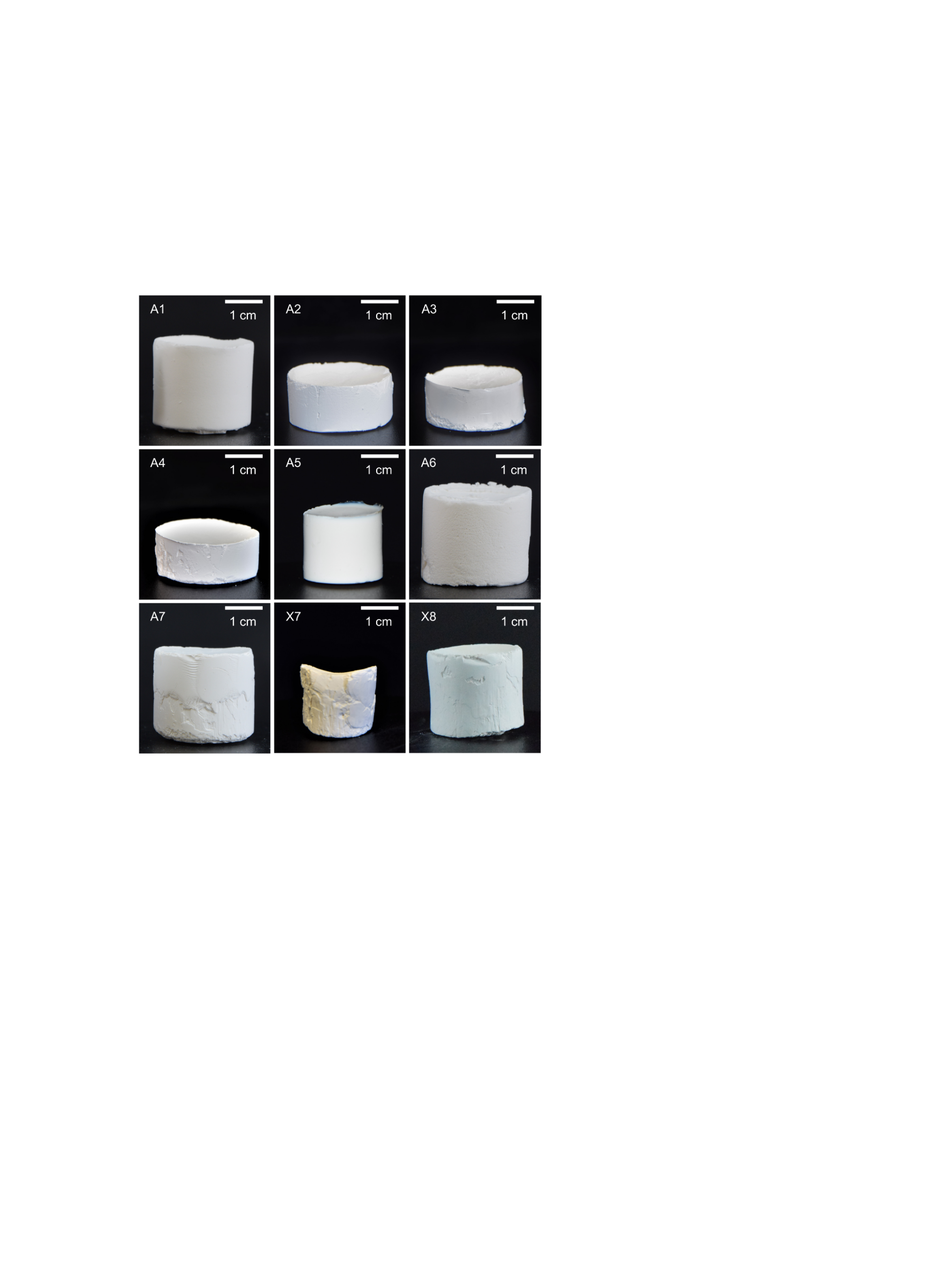


**Figure S8:** Photographs of TMBF aerogels and xerogels.

# Characterization of aerogels and xerogels

## ATR-IR Spectroscopy

Attenuated Total Reflection Infrared (ATR-IR) spectroscopy was performed on a Tensor 27 spectrometer equipped with a Platinum-ATR unit (A225/Qx, Bruker Optics GmbH & Co.KG, Ettlingen, Germany). Measurements were performed over a spectral range of 4000-400 cm^-1^ with a resolution of 4 cm^-1^. using 128 scans. The processing of raw data was conducted with OPUS 7.0 Software (Bruker Optics GmbH & Co.KG, Ettlingen, Germany) including subtraction of background measurements (64 scans) and correction of the baseline (concave rubberband method, 5 iterations, 64 baseline points). The spectral intensities were normalized using OriginPro 2023 software (Northampton, Massachusetts, USA).





**Figure S9:** ATR-IR spectrum of A1.





**Figure S10:** ATR-IR spectrum of A2.





**Figure S11:** ATR-IR spectrum of A3.





**Figure S12:** ATR-IR spectrum of A4.





**Figure S13:** ATR-IR spectrum of A5.





**Figure S14:** ATR-IR spectrum of A6.





**Figure S15:** ATR-IR spectrum of A7.





**Figure S16:** ATR-IR spectrum of X7.





**Figure S17:** ATR-IR spectrum of X8.

## ^13^C CP-MAS NMR

Solid state ^13^C Cross-Polarization Magic Angle Spinning Nuclear Magnetic Resonance (CP-MAS NMR) spectroscopy was performed by Müller NMR Laboratory (Trento University, Trento, Italy) using an AV400Avance WB spectrometer (Bruker BioSpin GmbH, Rheinstetten, Germany) equipped with a 4 mm double channel CP-MAS probe using a proton-decoupled cross-polarization pulse sequence at a ^13^C frequency of 100.48 MHz. The samples were packed in 4 mm zirconia rotors and measured at room temperature under air flow using spinning frequencies of 8 and 10 kHz for each sample. Adamantane (*δ* (CH_2_) = 38.5 ppm) was used as external secondary reference. Spectral intensities were normalized using OriginPro 2023 software (Northampton, Massachusetts, USA).


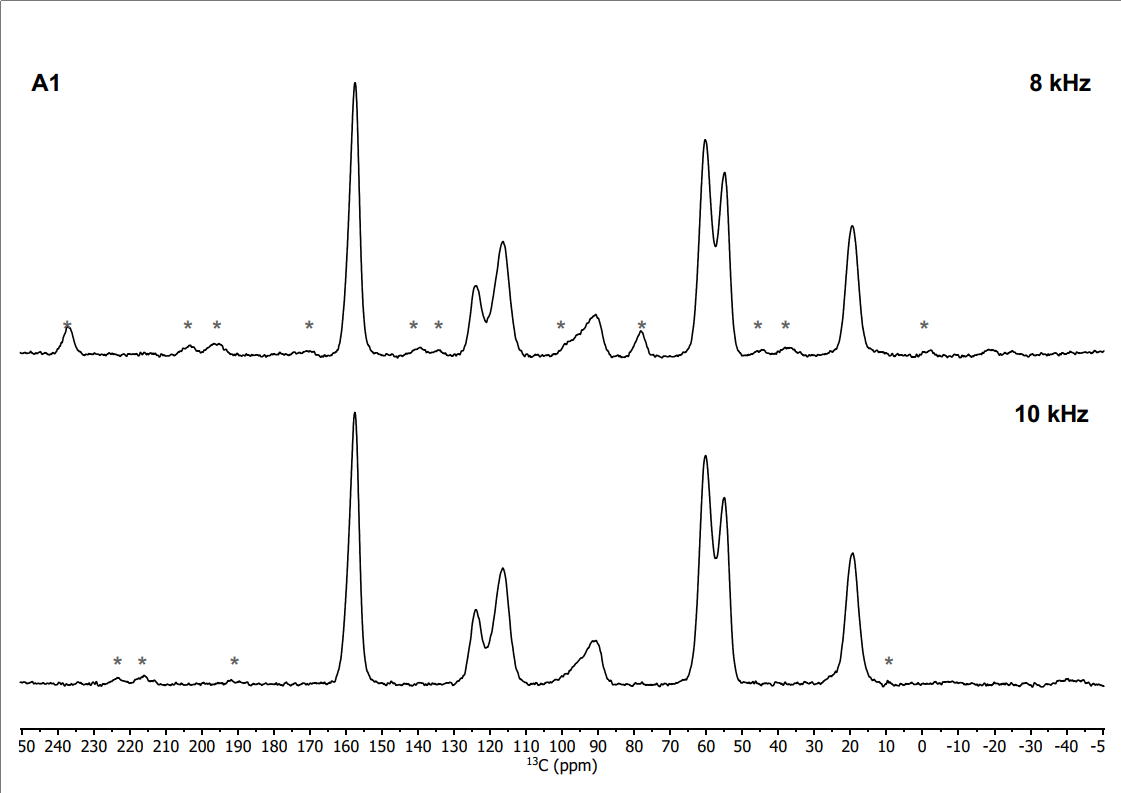


**Figure S18:** ^13^C-CP-MAS NMR spectra of sample A1 recorded with spinning frequencies of 8 kHz and 10 kHz. Spinning side bands are denoted as “*”.


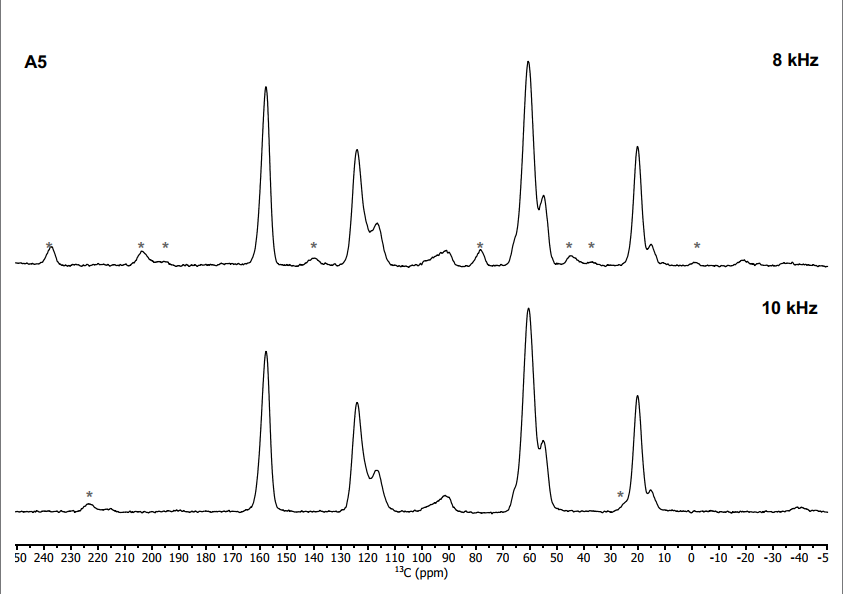


**Figure S19:** ^13^C-CP-MAS NMR spectra of sample A5 recorded with spinning frequencies of 8 kHz and 10 kHz. Spinning side bands are denoted as “*”.


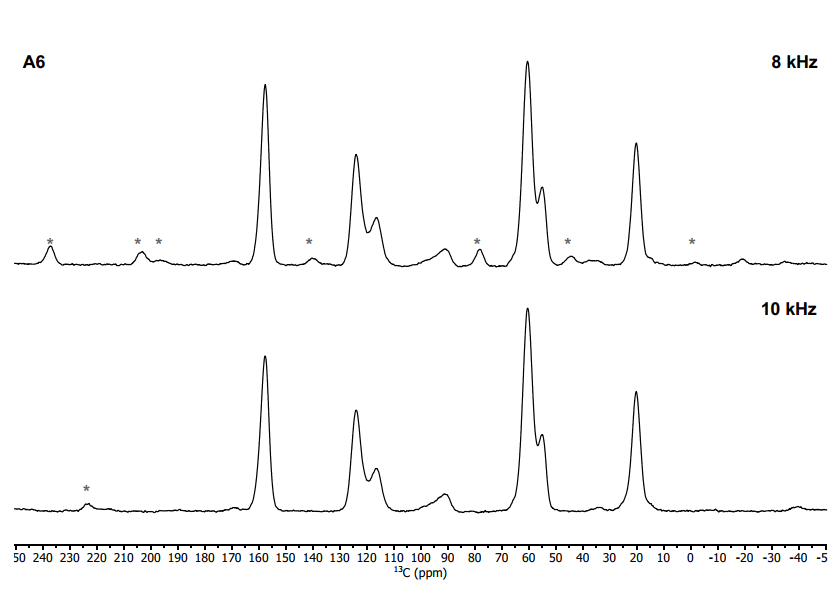


**Figure S20:** ^13^C-CP-MAS NMR spectra of sample A6 recorded with spinning frequencies of 8 kHz and 10 kHz. Spinning side bands are denoted as “*”.


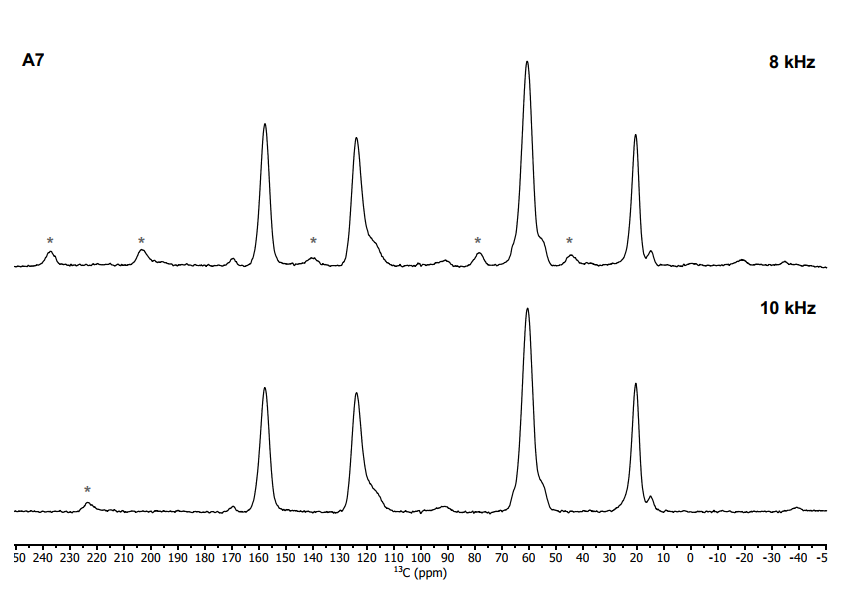


**Figure S21:** ^13^C-CP-MAS NMR spectra of sample A7 recorded with spinning frequencies of 8 kHz and 10 kHz. Spinning side bands are denoted as “*”.

## Determination of shrinkage

Using a digital caliper, both inner diameter of the synthesis mold (32.37 mm) and outer diameter of dry monolithic material were measured. The radial shrinkage of monoliths was determined as the ratio between these two quantities. A summary of measured diameters and calculated shrinkages are summarized in **Table S3**.

**Table S3:** Diameters and shrinkage of TMBF aerogels A1-A7 and xerogels X7 & X8.

| Sample | Diameter | | | | | Average  diameter | Shrinkage |
| --- | --- | --- | --- | --- | --- | --- | --- |
|  | 1 | | 2 | 3 | |  |  |
| A1 | 27.18 | 27.59 | | | 27.76 | 27.51 | 15 |
| A2 | 29.21 | 29.06 | | | 29.01 | 29.09 | 10 |
| A3 | 26.92 | 27.20 | | | 27.84 | 27.32 | 16 |
| A4 | 28.50 | 28.85 | | | 28.82 | 28.72 | 11 |
| A5 | 22.73 | 22.70 | | | 22.81 | 22.75 | 30 |
| A6 | 30.66 | 30.33 | | | 30.13 | 30.37 | 6.2 |
| A7 | 28.80 | 28.07 | | | 28.05 | 28.31 | 13 |
| X7 | 21.29 | 22.12 | | | 22.60 | 22.00 | 32 |
| X8 | 26.69 | 26.13 | | | 26.37 | 26.40 | 18 |

## Pycnometry and determination of porosity and total pore volume

Envelope densities were measured with a Geopyc 1360 sand pycnometer (Micromeritics GmbH, Unterschleissheim, Germany) using DRYFLO™ (Micromeritics GmbH, Unterschleissheim, Germany) as sand medium. For each measurement 10 cycles with a consolidation force of 5 N were performed. Skeletal densities were determined with an AccuPyc II 1340 helium pycnometer (Micromeritics GmbH, Unterschleissheim, Germany). The purge- and cycle fill pressure was set to 19.500 psig, and equilibration was ended when a change rate of 0.0050 psig/min was reached. Each sample was purged and measured 10 times. The results are listed in **Table S4**.

**Table S4:** Skeletal density measurements of TMBF aerogels A1-A7 and xerogels X7-X8.

| Cycle | A1 | A2 | A3 | A4 | A5 | A6 | A7 | X7 | X8 |
| --- | --- | --- | --- | --- | --- | --- | --- | --- | --- |
| 1 | 1.3179 | 1.2702 | 1.2899 | 1.3329 | 1.2847 | 1.3002 | 1.3143 | 1.2920 | 1.3175 |
| 2 | 1.3190 | 1.3061 | 1.2963 | 1.3473 | 1.2645 | 1.3347 | 1.3073 | 1.2959 | 1.3218 |
| 3 | 1.3264 | 1.3151 | 1.2732 | 1.3489 | 1.2863 | 1.3340 | 1.3004 | 1.2954 | 1.3277 |
| 4 | 1.3067 | 1.2934 | 1.3074 | 1.3486 | 1.2750 | 1.3397 | 1.3119 | 1.2960 | 1.3263 |
| 5 | 1.3232 | 1.3167 | 1.3022 | 1.3126 | 1.2628 | 1.3436 | 1.3096 | 1.2992 | 1.3217 |
| 6 | 1.3294 | 1.3166 | 1.3092 | 1.3201 | 1.2745 | 1.3484 | 1.3073 | 1.2936 | 1.3205 |
| 7 | 1.3245 | 1.2877 | 1.2686 | 1.3526 | 1.2779 | 1.3271 | 1.3166 | 1.2998 | 1.3235 |
| 8 | 1.3088 | 1.3174 | 1.2821 | 1.3271 | 1.2843 | 1.3441 | 1.3237 | 1.2974 | 1.3254 |
| 9 | 1.3102 | 1.3055 | 1.3073 | 1.3481 | 1.2629 | 1.3252 | 1.3358 | 1.2953 | 1.3225 |
| 10 | 1.3302 | 1.3027 | 1.2900 | 1.3085 | 1.2944 | 1.3463 | 1.3073 | 1.2959 | 1.3241 |
| Average | 1.3196 | 1.3031 | 1.2926 | 1.3347 | 1.2767 | 1.3343 | 1.3134 | 1.2961 | 1.3231 |
| St. deviation | 0.0082 | 0.0146 | 0.0138 | 0.0158 | 0.0103 | 0.0136 | 0.0096 | 0.0022 | 0.0028 |

The porosity of each sample was calculated using **Equation S1**, where *Φ*, *ρ*_e_ and *ρ*_s_ are the porosity, the envelope density and the skeletal density, respectively.

| $\Phi=\left( 1-\frac{\rho_{e}}{\rho_{s}} \right)\cdot100$ | Equation S1 |
| --- | --- |

The total pore volume *V*_p,total_ of each sample was calculated with **Equation S2**, where *ρ*_e_ and *ρ*_s_ are the envelope density the skeletal density, respectively.

| $P_{V,Total}=\frac{1}{\rho_{e}}-\frac{1}{\rho_{s}}$ | Equation S2 |
| --- | --- |

## Physisorption

Physisorption isotherms were measured on a 3Flex Physisorption Analyzer (Micromeritics GmbH, Unterschleissheim, Germany) at 77.3 K using N_2_ or ,Kr in case of small specific surface areas (< 5 m^2^/g), as gases. Relative pressure ranges of *p*/*p*^0^ = 0.0-1.0 and *p*/*p*^0^ = 0.0-0.2 have been used for N_2_ and Kr sorption, respectively. All samples were outgassed at 120 °C under vacuum conditions for 15 h, using a Smart VacPrep HIVAC apparatus (Micromeritics GmbH, Unterschleissheim, Germany). Due to the presence of low-pressure hysteresis, control experiments were performed under variation of the time for equilibration of relative pressures (**Figure S22**, a) and by measuring two consecutive N_2_ sorption isotherms for one sample (**Figure S22**, b).


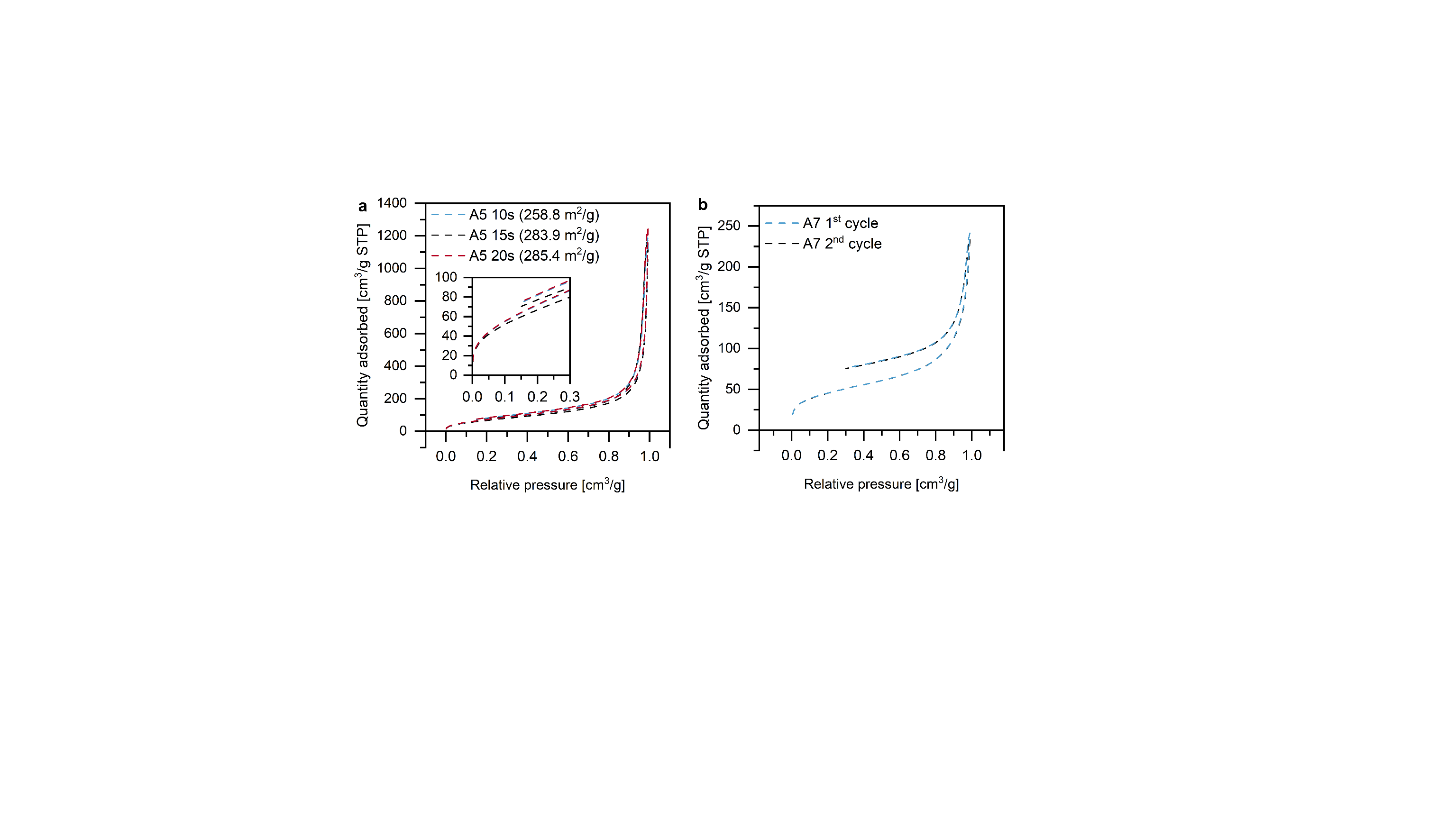


**Figure S22:** a) N_2_ sorption isotherms of sample A5 measured with equilibration intervals of 10 s, 15 s and 20 s. b) Two consecutive measurements of the N_2_ sorption isotherm of sample A7.

The persistence of the low-pressure hysteresis at higher equilibration time suggests that equilibrium pressure is reached in both experiments. Specific surface areas were calculated using the method described by *Brunauer*-*Emmett*-*Teller* (BET)***,*** following the IUPAC recommendations for the application of the BET method to porous materials. ^[33]^

## Scanning electron microscopy (SEM)

Scanning electron microscopy was performed on an ULTRA55 scanning electron microscope (Carl Zeiss AG, Oberkochen, Germany) using an operating voltage of 3-5 kV. A thin platinum layer was sputtered with a SCD 050 sputter-coater (BalTec AG, Pfäffikon, Switzerland) on all samples for 60-90 s with a current of 21 mA before measurements. Images were generally captured using a secondary electron (SE2) detector. Even though each sample was sputtered with Pt, some samples were easily electrostatically charged, which made capturing of SEM images challenging in such cases. Charging artifacts could, to some degree, be circumvented by using an angle selective back-scatter electron (AsB) or an immersion lens detector instead of a SE2 detector.


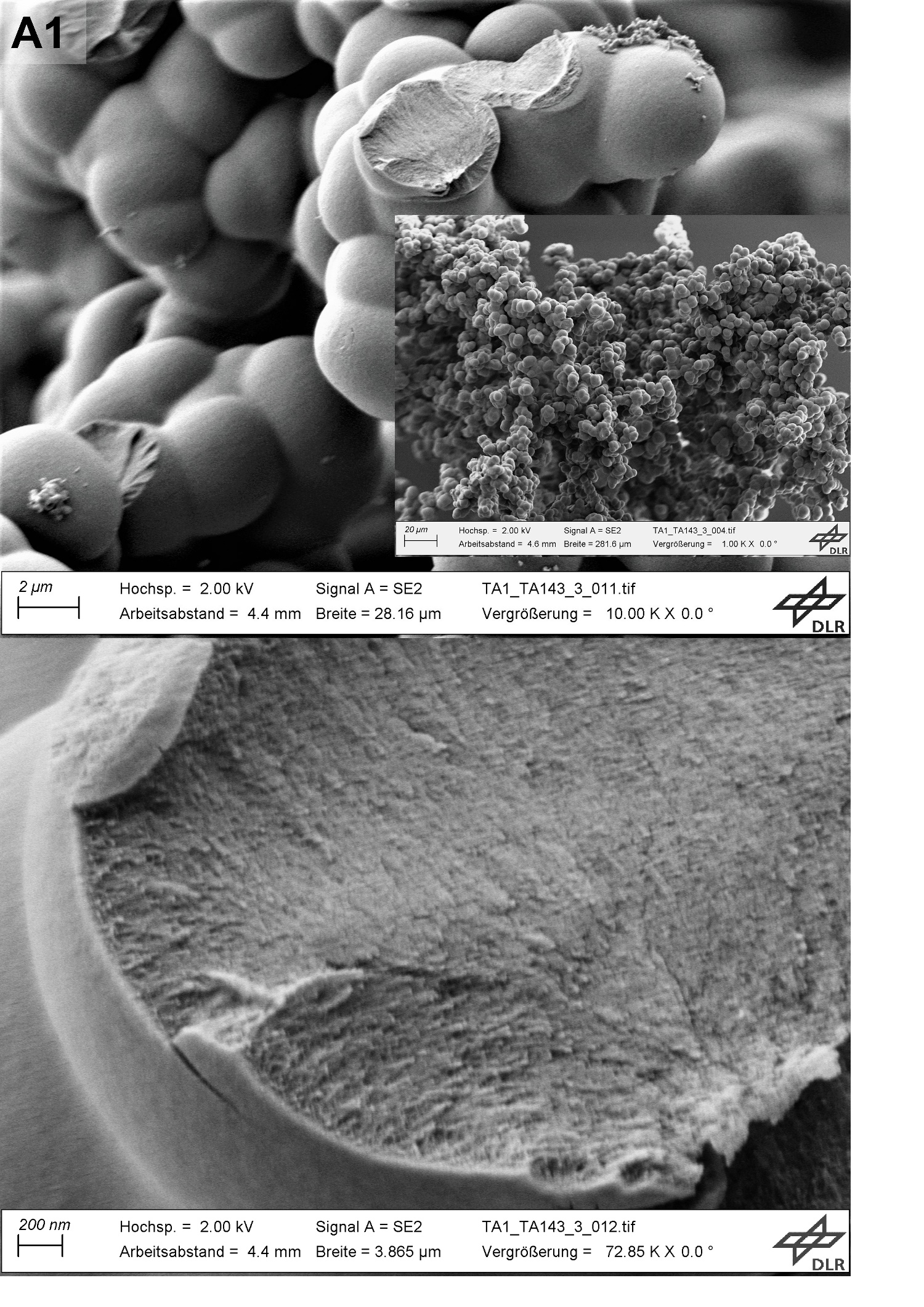


**Figure S23:** SEM images of A1.


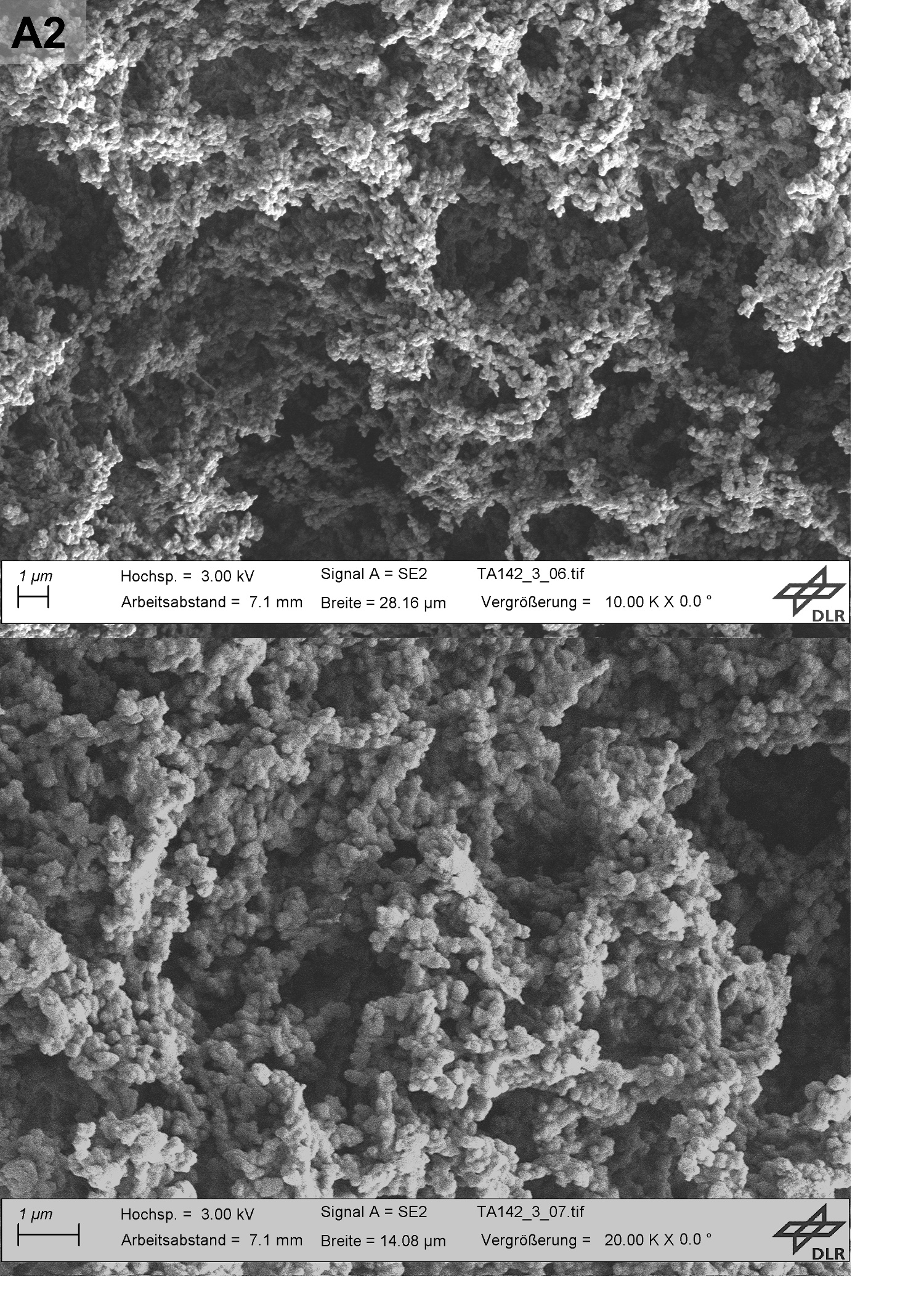


**Figure S24:** SEM images of A2.





**Figure S25:** SEM images of A3.


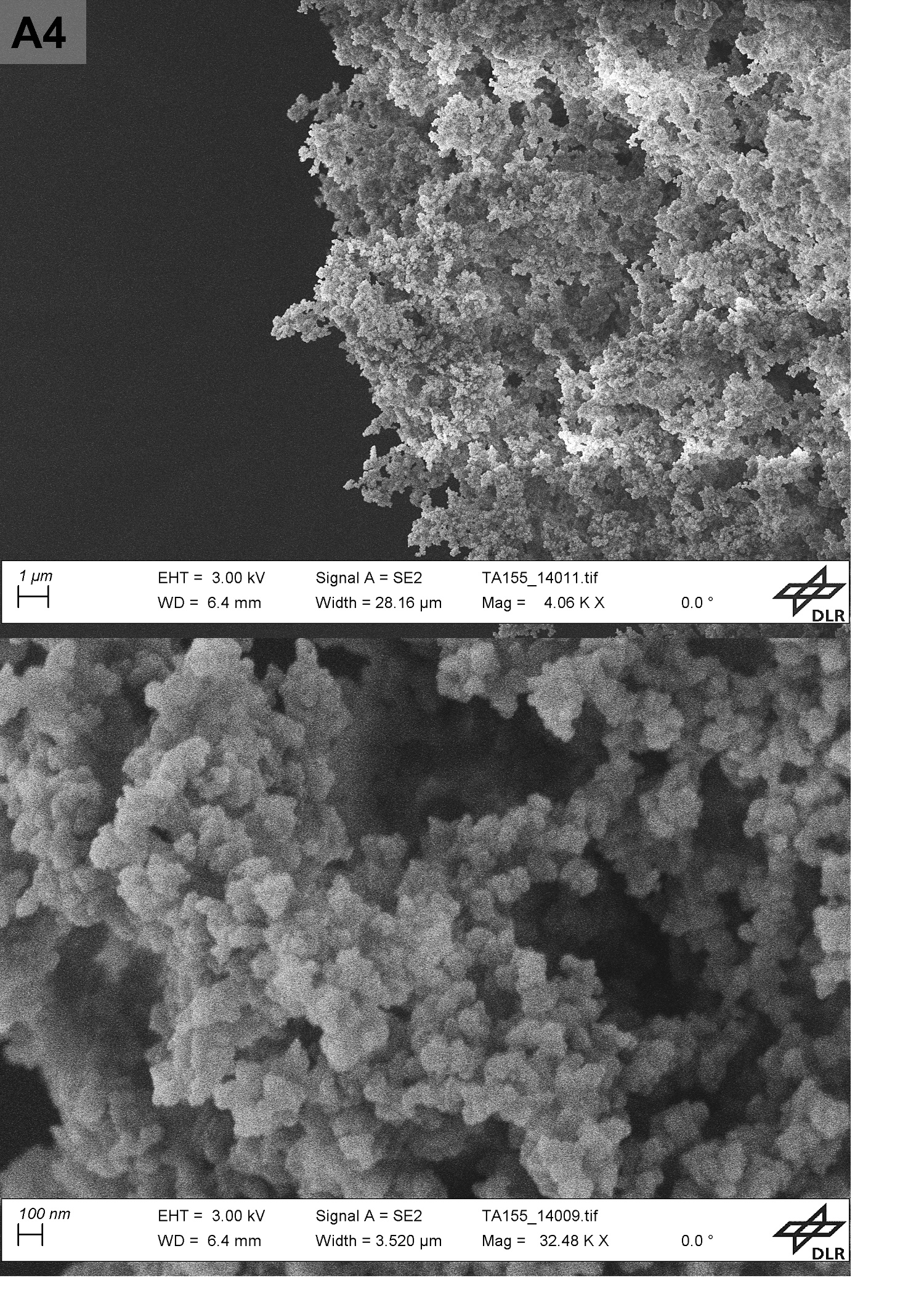


**Figure S26:** SEM images of A4.


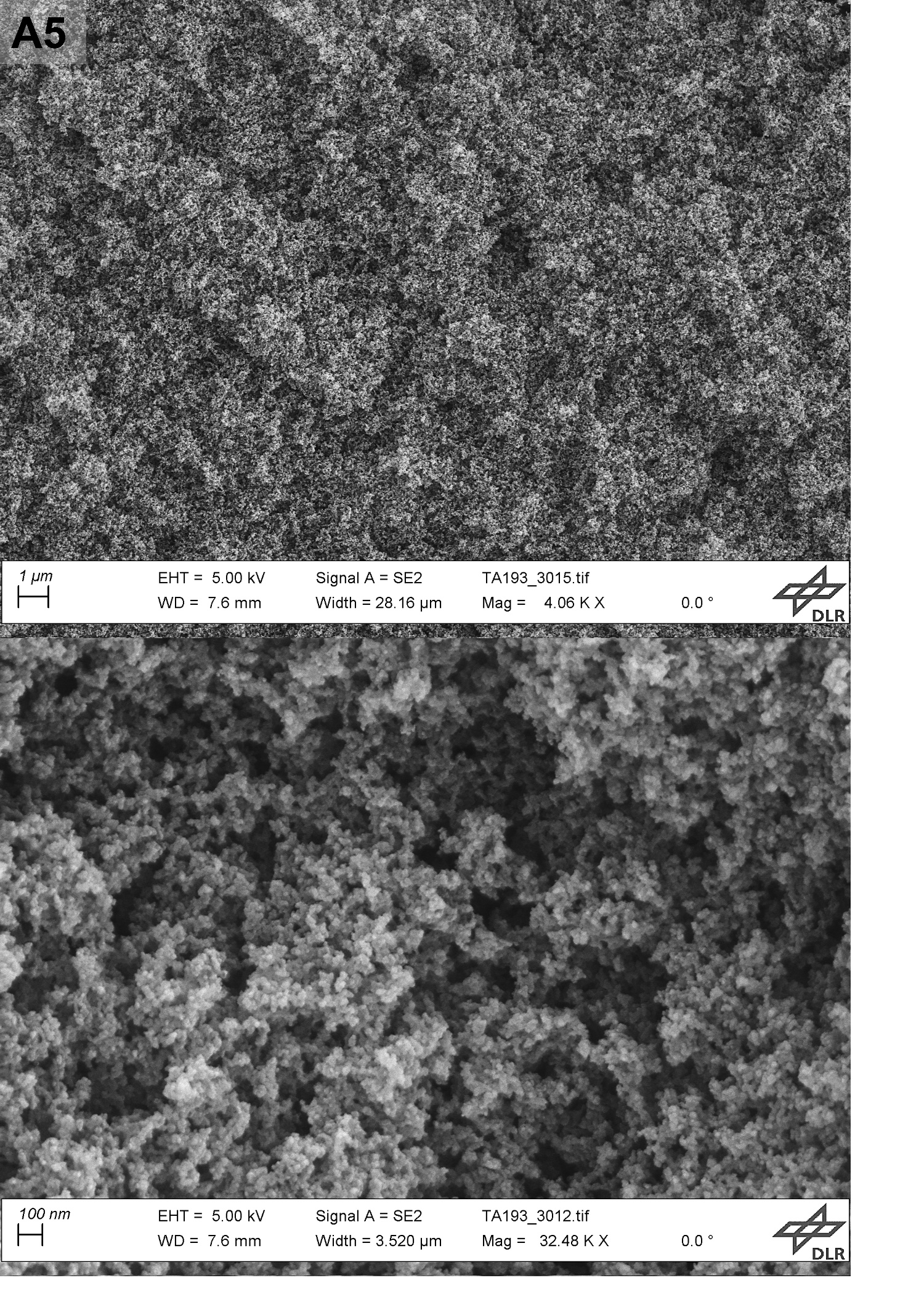


**Figure S27:** SEM images of A5.


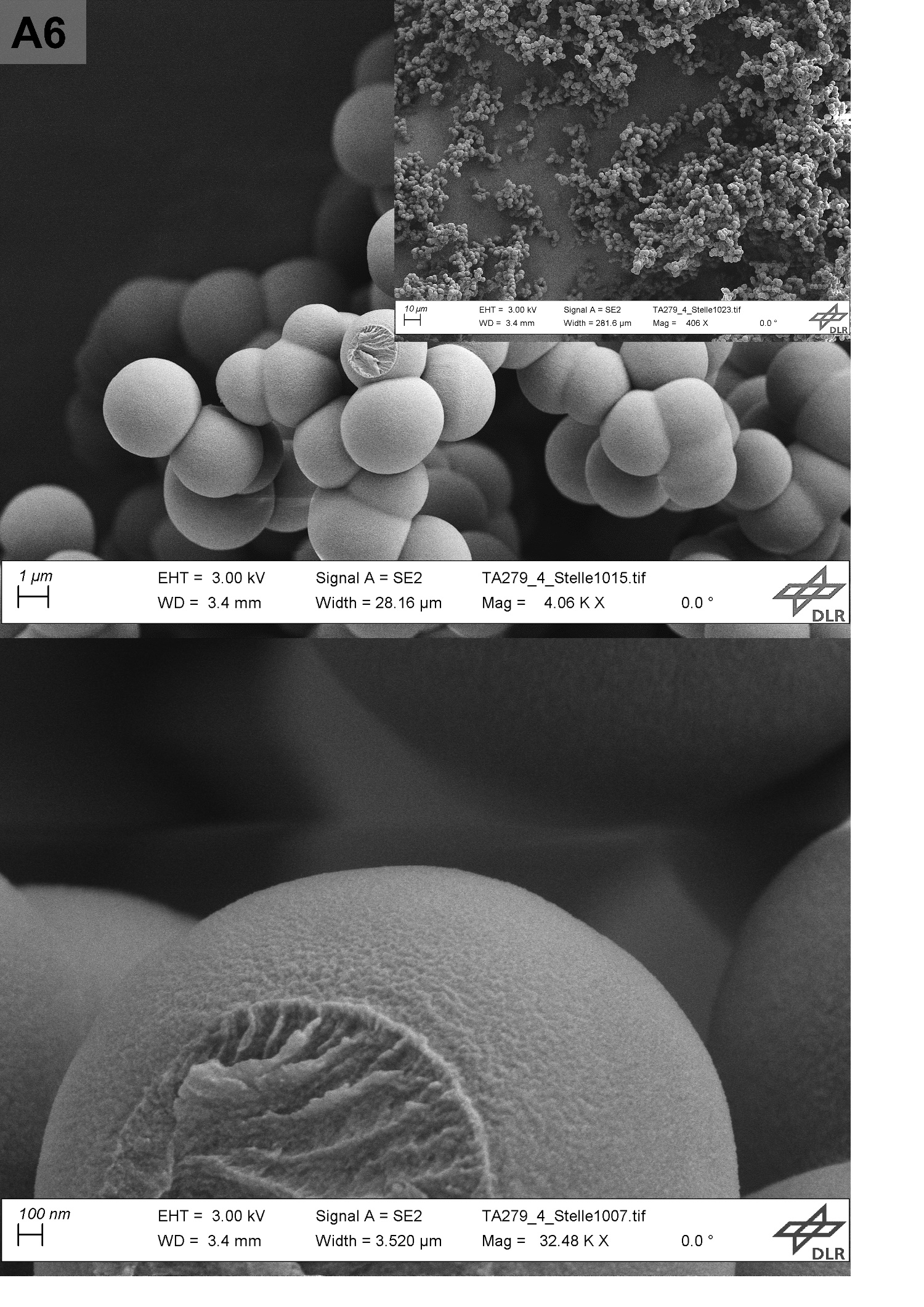


**Figure S28:** SEM images of A6.


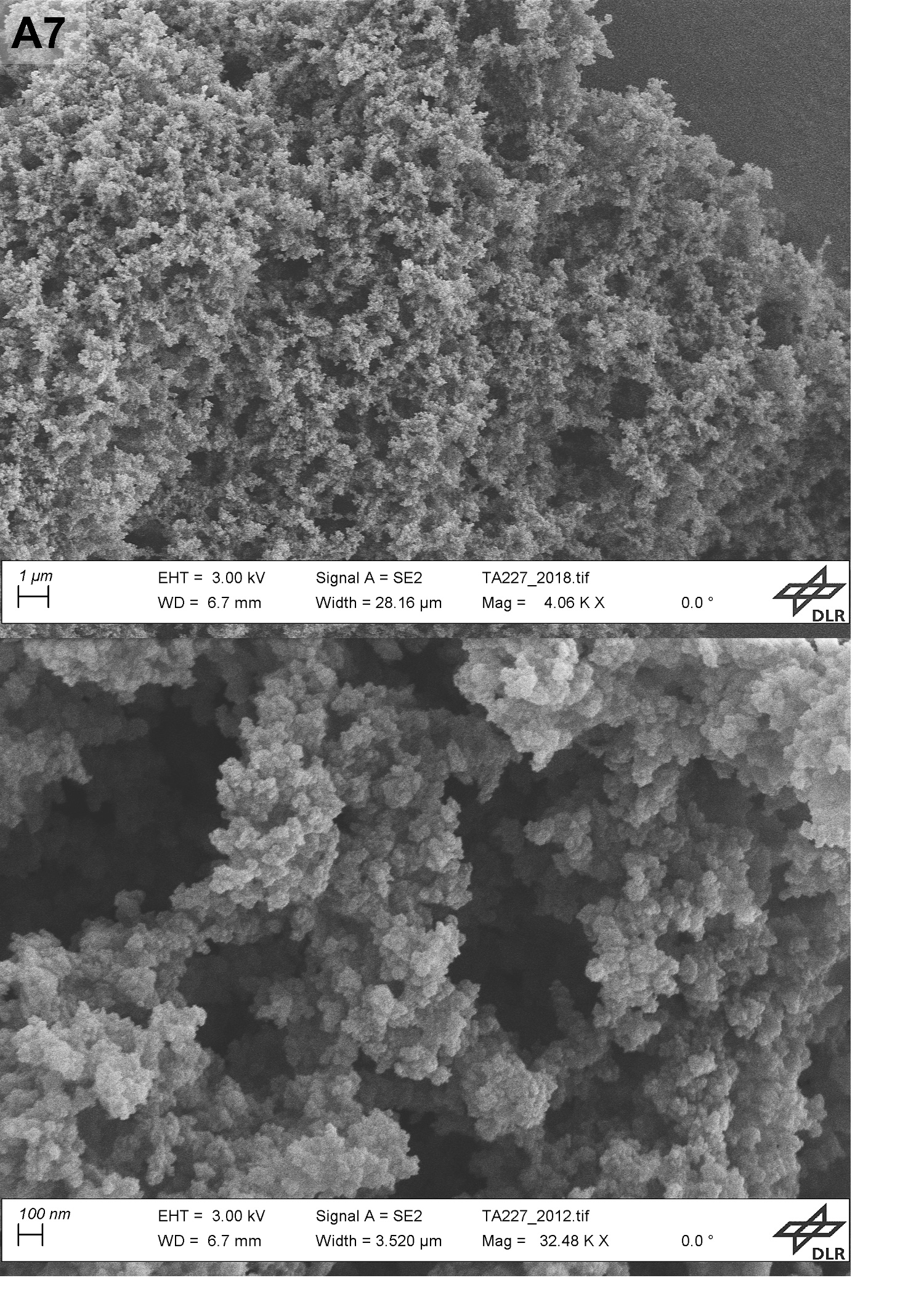


**Figure S29:** SEM images of A7.


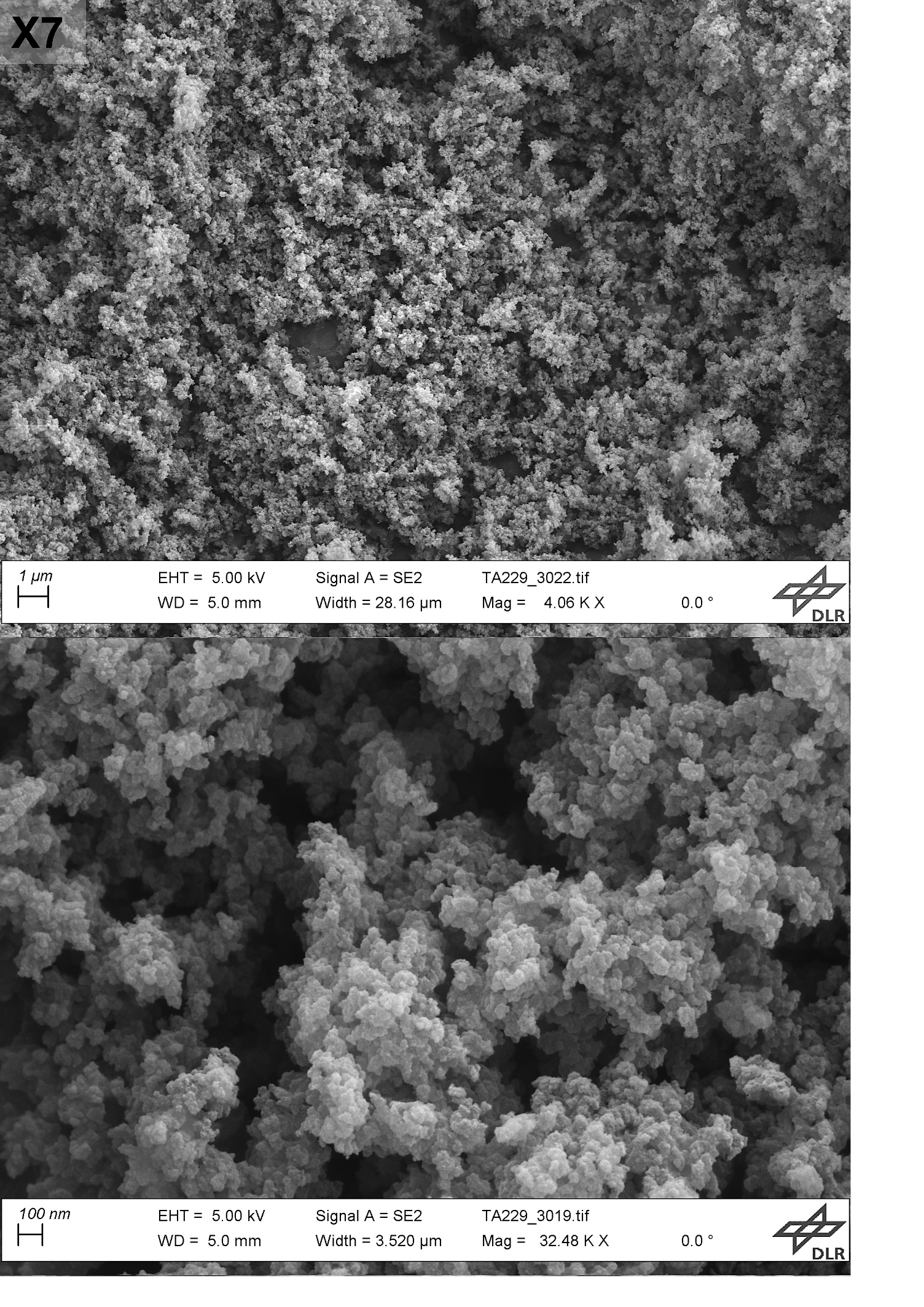


**Figure S30:** SEM images of X7.


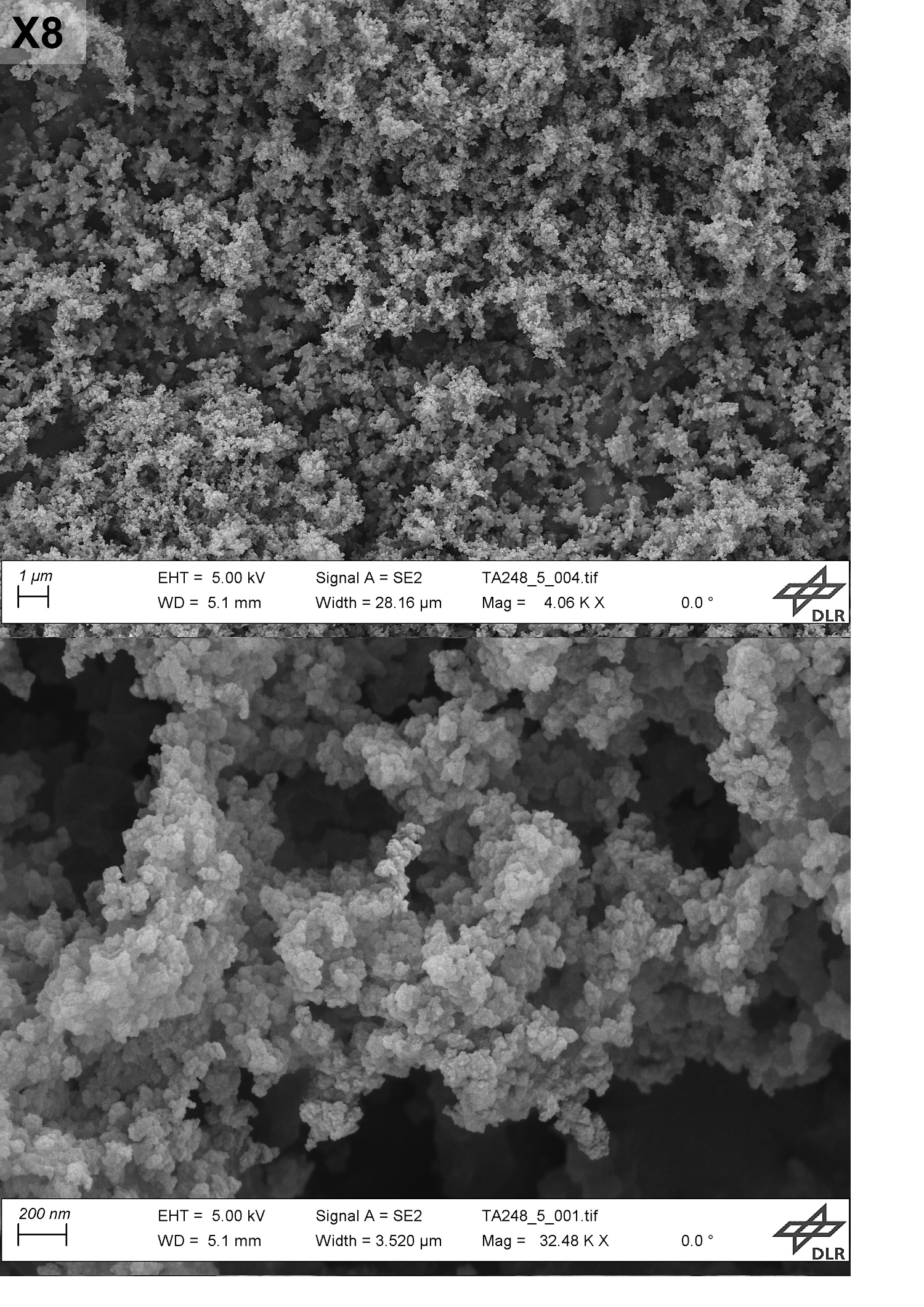


**Figure S31:** SEM images of X8.

## Wetting Behavior

Contact angles were determined by placing 5 μL of DI water on a sanded surface (120 grit) and taking an image with an USB microscope. The ImageJ *DropSnake* plugin^[48]^ was used to derive the contact angles. For each sample, contact angles were determined at three different spots and for both contact points of the droplet to cancel out local inhomogeneities.


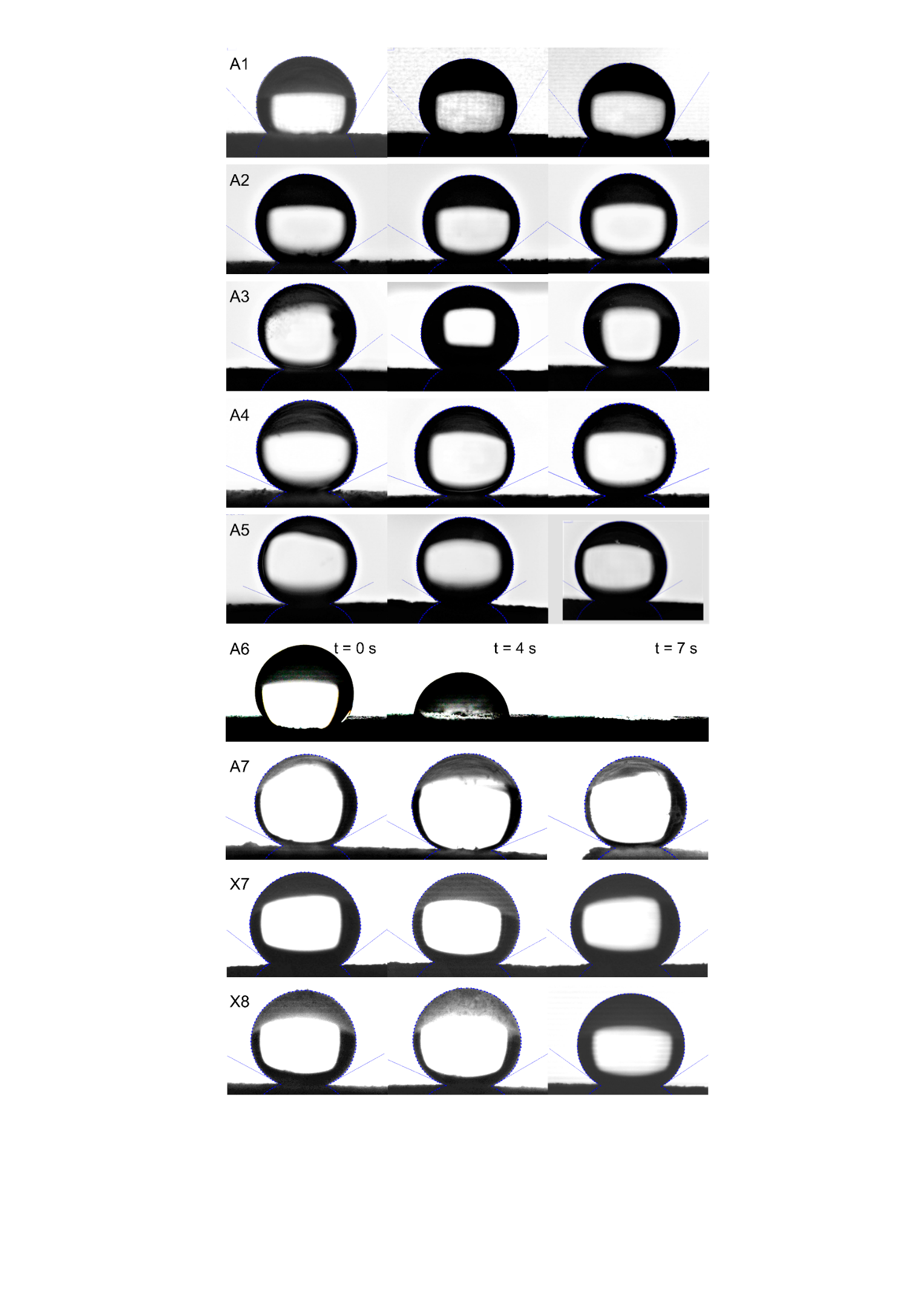


**Figure S32:** Water droplets on TMBF aerogels A1-A7 and xerogels X7-X8.

**Table S5:** Contact angle measurements for TMBF aerogels A1-A7 and xerogels X7-X8.

| Sample | Contact angle [°] | | | | | | Average |
| --- | --- | --- | --- | --- | --- | --- | --- |
|  | 1 | 2 | 3 | 4 | 5 | 6 |  |
| A1 | 132.8 | 132.6 | 133.1 | 128.4 | 130.6 | 122.8 | 130.1 ± 3.6 |
| A2 | 143.3 | 148.0 | 149.4 | 141.1 | 145.9 | 144.7 | 145.4 ± 2.8 |
| A3 | 153.1 | 141.3 | 145.8 | 145.1 | 150.5 | 145.0 | 146.8 ± 3.9 |
| A4 | 155.5 | 154.0 | 159.9 | 155.7 | 155.9 | 157.6 | 156.4 ± 1.9 |
| A5 | 156.8 | 154.0 | 151.2 | 147.2 | 149.2 | 147.9 | 151.1 ± 3.4 |
| A6 | - | - | - | - | - | - | - |
| A7 | 151.3 | 153.3 | 150.4 | 152.8 | 156.6 | 151.9 | 152.8 ± 1.9 |
| X7 | 143.8 | 140.8 | 147.2 | 149.3 | 142.3 | 142.5 | 144.3 ± 3.0 |
| X8 | 153.3 | 149.8 | 156.7 | 149.0 | 144.8 | 145.7 | 149.9 ± 4.1 |

## Thermal Stability

Thermogravimetric analyses (TGA) were performed on a STA449 F3 Jupiter apparatus comprising a SiC oven using PtRh20 crucibles (V = 85 μL) equipped with a lid and an Al_2_O_3_ liner (Netzsch-Gerätebau GmbH, Selb, Germany). The analytical process was conducted in an argon atmosphere and involved a starting temperature of 35 °C, a heating rate of 10 °C/min, and an end temperature of 600 °C.

## Thermal conductivity

For the determination of thermal conductivity, the transient plane source technique was applied using a HotDiskTP2500 apparatus (HotDisk AB, Sweden).^[49]^ A Kapton-insulated sensor with a radius of 6.4 mm (model 5501) was used. Measurements were performed using heating power of 10 mW for periods of 80 seconds. A total of seven measurements was recorded for each sample in order to allow for statistical analysis, and idle periods of at least 120 min were applied in between measurements in order to allow for equilibration of sample temperatures.

**Table S6:** Summary of thermal conductivity measurements for A1.

|  | Thermal conductivity [W/mK] | Probing depth [mm] | Temperature increase [K] | *Total/ Temperature increase [K]* |
| --- | --- | --- | --- | --- |
| A1_1 | 0.0429 | 7.49 | 1.132 | 9.337 |
| A1_2 | 0.0431 | 7.53 | 1.132 | 9.443 |
| A1_3 | 0.0388 | 6.67 | 1.201 | 9.491 |
| A1_4 | 0.0367 | 6.35 | 1.242 | 9.579 |
| A1_5 | 0.0418 | 7.53 | 1.166 | 9.385 |
| A1_6 | 0.0399 | 7.29 | 1.202 | 9.440 |
| A1_7 | 0.0375 | 6.81 | 1.250 | 9.505 |
| Average | 0.0401 |  |  |  |
| St. dev. | 0.0024 |  |  |  |





**Figure S33:** Transient- and residual plots of A1.

**Table S7:** Summary of thermal conductivity measurements for A2.

|  | Thermal conductivity [W/mK] | Probing depth [mm] | Temperature increase [K] | *Total/ Temperature increase [K]* |
| --- | --- | --- | --- | --- |
| A2_1 | 0.0437 | 7.40 | 1.016 | 9.383 |
| A2_2 | 0.0432 | 7.37 | 1.026 | 9.381 |
| A2_3 | 0.0443 | 7.54 | 1.009 | 9.384 |
| A2_4 | 0.0440 | 7.51 | 1.011 | 9.316 |
| A2_5 | 0.0438 | 7.42 | 1.015 | 9.385 |
| A2_6 | 0.0428 | 7.35 | 1.033 | 9.306 |
| A2_7 | 0.0451 | 7.93 | 1.009 | 9.399 |
| Average | 0.0439 |  |  |  |
| St. Dev. | 0.0007 |  |  |  |





**Figure S34:** Transient- and residual plots of A2.

**Table S8:** Summary of thermal conductivity measurements for A5.

|  | Thermal conductivity [W/mK] | Probing depth [mm] | Temperature increase [K] | *Total/ Temperature increase [K]* |
| --- | --- | --- | --- | --- |
| A5_1 | 0.0442 | 6.68 | 1.144 | 9.158 |
| A5_2 | 0.0431 | 6.45 | 1.155 | 9.191 |
| A5_3 | 0.0444 | 6.69 | 1.140 | 9.174 |
| A5_4 | 0.0431 | 6.49 | 1.161 | 9.196 |
| A5_5 | 0.0437 | 6.57 | 1.152 | 9.176 |
| A5_6 | 0.0446 | 6.74 | 1.139 | 9.182 |
| A5_7 | 0.0432 | 6.47 | 1.153 | 9.241 |
| Average | 0.0438 |  |  |  |
| St. Dev. | 0.0006 |  |  |  |





**Figure S35:** Transient- and residual plots of A5.

**Table S9:** Summary of thermal conductivity measurements for A7.

|  | Thermal conductivity [W/mK] | Probing depth [mm] | Temperature increase [K] | *Total/ Temperature increase [K]* |
| --- | --- | --- | --- | --- |
| A7_1 | 0.0344 | 6.72 | 1.254 | 9.677 |
| A7_2 | 0.0344 | 6.60 | 1.245 | 9.626 |
| A7_3 | 0.0346 | 6.62 | 1.236 | 9.651 |
| A7_4 | 0.0346 | 6.65 | 1.239 | 9.625 |
| A7_5 | 0.0345 | 6.64 | 1.235 | 9.644 |
| A7_6 | 0.0344 | 6.54 | 1.234 | 9.653 |
| A7_7 | 0.0346 | 6.63 | 1.236 | 9.637 |
| Average | 0.0345 |  |  |  |
| St. Dev. | 0.0001 |  |  |  |





**Figure S36:** Transient- and residual plots of A7.

**Table S10:** Summary of thermal conductivity measurements for X8.

|  | Thermal conductivity [W/mK] | Probing depth [mm] | Temperature increase [K] | *Total/ Temperature increase [K]* |
| --- | --- | --- | --- | --- |
| X8_1 | 0.0348 | 6.58 | 1.035 | 9.592 |
| X8_2 | 0.0344 | 6.40 | 1.042 | 9.561 |
| X8_3 | 0.0344 | 6.34 | 1.036 | 9.567 |
| X8_4 | 0.0347 | 6.46 | 1.031 | 9.556 |
| X8_5 | 0.0345 | 6.45 | 1.038 | 9.574 |
| X8_6 | 0.0346 | 6.38 | 1.030 | 9.540 |
| X8_7 | 0.0344 | 6.44 | 1.039 | 9.544 |
| Average | 0.0345 |  |  |  |
| St. Dev. | 0.0002 |  |  |  |





**Figure S37:** Transient- and residual plots of X8.

## Mechanical properties - Compression tests

Before testing, specimens were sanded using 200 grid sanding paper. Quasi-static uniaxial compression tests were performed using an Instron 5566A universal testing machine (Instron GmbH, Darmstadt, Germany) equipped with 1kN- or 5kN-load cells (Instron GmbH, Darmstadt, Germany). A displacement rate of 1mm/min was applied to the specimens. Evaluation of the data was based on the procedures outlined in ISO844:2021, Method A.^[50]^ Tests were generally performed in triplicate fashion using cylindrical samples. Sample X7, however, was measured in duplicate fashion and in the shape of rectangular prism.

**Table S11:** Summary of mechanical properties of sample A1.

| **A1** (cylindrical) | a | b | c | average |
| --- | --- | --- | --- | --- |
| maximum force of load cell/ kN | 5 | 1 | 5 | - |
| height / mm | 17.3 | 18.8 | 17.0 | 17.7 ± 0.8 |
| diameter / mm | 27.9 | 28.0 | 28.3 | 28.1 ± 0.2 |
| nominal compressive modulus of elasticity *E*_C_ / MPa | 3.30 | 4.25 | 2.65 | 3.40 ± 0.65 |
| nominal relative deformation *ε*_c_ / % | 3.15 | 3.16 | 4.73 | 3.68 ± 0.74 |
| compressive stress at 10% nominal relative deformation *σ*_10_ / MPa | 0.227 | 0.227 | 0.225 | 0.227 ± 0.001 |
| compressive strength *σ*_m_ / MPa | 0.234 | 0.259 | 0.295 | 0.262 ± 0.025 |
| nominal relative deformation (corresponding to *σ*_m_) *ε_C_*_m_ / % | 12.3 | 22.0 | 31.9 | 22.0 ± 8.0 |

**Figure S38:** Stress strain curves of 3 monoliths of sample A1.

**Table S12:** Summary of mechanical properties of sample A2.

| **A2** (cylindrical) | a | b | c | average |
| --- | --- | --- | --- | --- |
| maximum force of load cell/ kN | 5 | 5 | 5 | - |
| height / mm | 20.0 | 19.0 | 19.0 | 19.3 ± 0.5 |
| diameter / mm | 29.2 | 29.2 | 29.2 | 29.2 ± 0.0 |
| nominal compressive modulus of elasticity *E*_C_ / MPa | 4.29 | 2.54 | 3.68 | 3.50 ± 0.72 |
| nominal relative deformation *ε*_c_ / % | 2.18 | 3.44 | 2.53 | 2.72 ± 0.53 |
| compressive stress at 10% nominal relative deformation *σ*_10_ / MPa | 0.202 | 0.193 | 0.206 | 0.200 ± 0.005 |
| compressive strength *σ*_m_ / MPa | no yield point | | | |
| nominal relative deformation (corresponding to *σ*_m_) *ε_C_*_m_ / % |  |  |  |  |

**Figure S39:** Stress strain curves of 3 monoliths of sample A2.

**Table S13:** Summary of mechanical properties of sample A5.

| **A5** (cylindrical) | a | b | c | average |
| --- | --- | --- | --- | --- |
| maximum force of load cell/ kN | 1 | 5 | 5 | - |
| height / mm | 17.2 | 17.0 | 16.2 | 16.8 ± 0.4 |
| diameter / mm | 22.2 | 22.2 | 22.2 | 22.2 ± 0.0 |
| nominal compressive modulus of elasticity *E*_C_ / MPa | 23.1 | 15.5 | 24.5 | 21.0 ± 4.0 |
| nominal relative deformation *ε*_c_ / % | 6.90 | 3.16 | 4.73 | 4.93 ± 1.54 |
| compressive stress at 10% nominal relative deformation *σ*_10_ / MPa | 1.58 | 1.39 | 1.60 | 1.52 ± 0.09 |
| compressive strength *σ*_m_ / MPa |  | 2.95 | 4.53 | 3.74 ± 0.79 |
| nominal relative deformation (corresponding to *σ*_m_) *ε_C_*_m_ / % |  | 28.4 | 37.0 | 32.7 ± 4.3 |

**Figure S40:** Stress strain curves of 3 monoliths of sample A5.

**Table S14:** Summary of mechanical properties of sample A7.

| **A7** (cylindrical) | a | b | c | average |
| --- | --- | --- | --- | --- |
| maximum force of load cell/ kN | 5 | 5 | 5 | - |
| height / mm | 21.3 | 20.0 | 21.0 | 20.8 ± 0.6 |
| diameter / mm | 27.5 | 27.2 | 27.4 | 27.4 ± 0.1 |
| nominal compressive modulus of elasticity *E*_C_ / MPa | 5.19 | 3.14 | 5.34 | 4.55 ± 1.00 |
| nominal relative deformation *ε*_c_ / % | 2.44 | 3.68 | 2.50 | 2.88 ± 0.57 |
| compressive stress at 10% nominal relative deformation *σ*_10_ / MPa | 0.315 | 0.251 | 0.308 | 0.291 ± 0.029 |
| compressive strength *σ*_m_ / MPa | 0.355 | 0.277 | 0.399 | 0.344 ± 0.051 |
| nominal relative deformation (corresponding to *σ*_m_) *ε_C_*_m_ / % | 14.0 | 12.4 | 18.9 | 15.1 ± 2.8 |

**Figure S41:** Stress strain curves of 3 monoliths of sample A7.

**Table S15:** Summary of mechanical properties of sample X7.

| **X7** (rectangular prism) | a | b | average |
| --- | --- | --- | --- |
| maximum force of load cell/ kN | 1 | 1 | - |
| height / mm | 6.6 | 9.3 | 7.9 ± 1.3 |
| length / mm | 14.2 | 15.9 | 15.0 ± 0.8 |
| width / mm | 11.3 | 8.4 | 9.9 ± 1.4 |
| nominal compressive modulus of elasticity *E*_C_ / MPa | 19.4 | 18.3 | 18.9 ± 0.6 |
| nominal relative deformation *ε*_c_ / % | 5.82 | 4.58 | 5.20 ± 0.62 |
| compressive stress at 10% nominal relative deformation *σ*_10_ / MPa | 1.76 | 1.58 | 1.67 ± 0.09 |
| compressive strength *σ*_m_ / MPa | 2.17 | 2.15 | 2.16 ± 0.01 |
| nominal relative deformation (corresponding to *σ*_m_) *ε_C_*_m_ / % | 14.8 | 21.8 | 18.3 ± 3.5 |

**Figure S42:** Stress strain curves of 2 monoliths of sample X7.

**Table S16:** Summary of mechanical properties of sample X8.

| **X8** (cylindrical) | a | b | c | average |
| --- | --- | --- | --- | --- |
| maximum force of load cell/ kN | 5 | 5 | 5 | - |
| height / mm | 18.9 | 18.4 | 18.3 | 18.5 ± 0.3 |
| diameter / mm | 25.0 | 25.5 | 25.3 | 25.3 ± 0.2 |
| nominal compressive modulus of elasticity *E*_C_ / MPa | 10.2 | 7.42 | 9.77 | 9.12 ± 1.21 |
| nominal relative deformation *ε*_c_ / % | 2.09 | 3.18 | 2.07 | 2.44 ± 0.52 |
| compressive stress at 10% nominal relative deformation *σ*_10_ / MPa | 0.609 | 0.558 | 0.610 | 0.592 ± 0.024 |
| compressive strength *σ*_m_ / MPa | 0.715 | 0.631 | 0.855 | 0.734 ± 0.092 |
| nominal relative deformation (corresponding to *σ*_m_) *ε_C_*_m_ / % | 14.6 | 13.7 | 20.9 | 16.4 ± 3.2 |

**Figure S43:** Stress strain curves of 3 monoliths of sample X8.

# References

[36] M. Thommes, K. Kaneko, A. V. Neimark, J. P. Olivier, F. Rodriguez-Reinoso, J. Rouquerol, K. S. W. Sing, *Pure Appl. Chem.* **2015**, *87*, 1051.

[47] A. Bueno, I. Selmer, R. S.P, P. Gurikov, W. Lölsberg, D. Weinrich, M. Fricke, I. Smirnova, *Ind. Eng. Chem. Res.* **2018**, *57*, 8698-8707.

[48] A. F. Stalder, G. Kulik, D. Sage, L. Barbieri, P. Hoffmann, *Colloids Surf., A* **2006**, *286*, 92-103.

[49] M. Gustavsson, E. Karawacki, S. E. Gustafsson, *Rev. Sci. Instrum.* **1994**, *65*, 3856-3859.

[50] International Organization for Standardization (ISO), *ISO 844:2021 Rigid cellular plastics - Determination of compression properties,* **2021**, Vernier, Switzerland.
